# The autophagic response to *Staphylococcus aureus* provides an intracellular niche in neutrophils

**DOI:** 10.1080/15548627.2020.1739443

**Published:** 2020-03-15

**Authors:** Tomasz K. Prajsnar, Justyna J. Serba, Bernice M. Dekker, Josie F. Gibson, Samrah Masud, Angeleen Fleming, Simon A. Johnston, Stephen A. Renshaw, Annemarie H. Meijer

**Affiliations:** a Bateson Centre and Department of Infection, Immunity and Cardiovascular Disease, University of Sheffield, Sheffield, UK; b Institute of Biology Leiden, Faculty of Science, Leiden University, Leiden, The Netherlands; c Krebs Institute and Department of Molecular Biology and Biotechnology, University of Sheffield, Sheffield, UK; d Department of Physiology, Development and Neuroscience, University of Cambridge, Cambridge, UK

**Keywords:** Autophagy, lc3-associated phagocytosis (LAP), NADPH oxidase, neutrophil, ROS, *Staphylococcus aureus*, zebrafish

## Abstract

*Staphylococcus aureus* is a major human pathogen causing multiple pathologies, from cutaneous lesions to life-threatening sepsis. Although neutrophils contribute to immunity against *S. aureus*, multiple lines of evidence suggest that these phagocytes can provide an intracellular niche for staphylococcal dissemination. However, the mechanism of neutrophil subversion by intracellular *S. aureus* remains unknown. Targeting of intracellular pathogens by macroautophagy/autophagy is recognized as an important component of host innate immunity, but whether autophagy is beneficial or detrimental to *S. aureus*-infected hosts remains controversial. Here, using larval zebrafish, we showed that the autophagy marker Lc3 rapidly decorates *S. aureus* following engulfment by macrophages and neutrophils. Upon phagocytosis by neutrophils, Lc3-positive, non-acidified spacious phagosomes are formed. This response is dependent on phagocyte NADPH oxidase as both *cyba/p22phox* knockdown and diphenyleneiodonium (DPI) treatment inhibited Lc3 decoration of phagosomes. Importantly, NADPH oxidase inhibition diverted neutrophil *S. aureus* processing into tight acidified vesicles, which resulted in increased host resistance to the infection. Some intracellular bacteria within neutrophils were also tagged by Sqstm1/p62-GFP fusion protein and loss of Sqstm1 impaired host defense. Together, we have shown that intracellular handling of *S. aureus* by neutrophils is best explained by Lc3-associated phagocytosis (LAP), which appears to provide an intracellular niche for bacterial pathogenesis, while the selective autophagy receptor Sqstm1 is host-protective. The antagonistic roles of LAP and Sqstm1-mediated pathways in *S. aureus*-infected neutrophils may explain the conflicting reports relating to anti-staphylococcal autophagy and provide new insights for therapeutic strategies against antimicrobial-resistant *Staphylococci*.

**Abbreviations:** ATG: autophagy related; CFU: colony-forming units; CMV: cytomegalovirus; Cyba/P22phox: cytochrome b-245, alpha polypeptide; DMSO: dimethyl sulfoxide; DPI: diphenyleneiodonium; EGFP: enhanced green fluorescent protein; GFP: green fluorescent protein; hpf: hours post-fertilization; hpi: hours post-infection; Irf8: interferon regulatory factor 8; LAP: LC3-associated phagocytosis; lyz: lysozyme; LWT: london wild type; Map1lc3/Lc3: microtubule-associated protein 1 light chain 3; NADPH oxidase: nicotinamide adenine dinucleotide phosphate oxidase; RFP: red fluorescent protein; ROS: reactive oxygen species; RT-PCR: reverse transcriptase polymerase chain reaction; Sqstm1/p62: sequestosome 1; Tg: transgenic; TSA: tyramide signal amplification.

## Introduction

*Staphylococcus aureus* is a highly successful human pathogen causing a wide range of diseases [1]. This microorganism is a leading cause of fatal bacteremia, with mortality rates reaching 30% [2] making multidrug resistance a particular concern [3]. Current therapeutic strategies to treat antimicrobial-resistant staphylococcal infections are becoming limited, and despite multiple attempts, there is still no vaccine available [4].

Although traditionally considered as an extracellular pathogen, accumulating evidence suggests that *S. aureus* is not only capable of inducing phagocyte lysis but also able to survive within professional phagocytes, such as macrophages [5] and neutrophils [6]. Neutrophils, although shown to play a role in immunity against *S. aureus* [7], can also provide an intracellular niche for staphylococcal dissemination or persistence [8–11]. However, little is known of how *S. aureus* can subvert host cells to avoid phagocyte killing. Therefore, there is a need for a better understanding of the interactions of intracellular *S. aureus* with phagocytes to develop therapies based on immunomodulatory approaches [12].

Autophagy is the evolutionarily-conserved intracellular degradation pathway by which eukaryotic cells scavenge their cytoplasmic contents through sequestration into a nascent phagophore whose edges subsequently fuse to form a double membrane-surrounded vesicle called the autophagosome and which then fuses with the lysosome for degradation [13]. MAP1LC3/LC3 (microtubule associated protein 1 light chain 3) is an autophagosomal marker decorating membrane phagophores during elongation and in the resulting autophagosomes. In addition to nutrient-recycling functions, targeting of intracellular pathogens by the autophagic machinery has become recognized as an important component of host innate immunity in a process called xenophagy. This selective degradation requires the use of ubiquitin receptors such as SQSTM1/p62 (sequestosome 1). However, multiple intracellular pathogens such as *Mycobacterium tuberculosis, Salmonella enterica*, or *Listeria monocytogenes* have evolved strategies to inhibit or subvert the autophagic response [14].

LC3-associated phagocytosis (LAP) is a recently described process that is similar to, but functionally and molecularly distinct from, canonical autophagy, which lacks the formation of the characteristic double-membrane autophagosome [15,16]. In this pathway, the lipidated form of LC3, which is directly coupled to the phagosomal membrane, decorates the bacteria-containing single-membrane phagosomes. This process requires the core autophagy machinery responsible for LC3 conjugation to lipids, such as ATG5 (autophagy related 5), but does not require early events of autophagosome initiation such as ULK1 (unc-51 like autophagy activating kinase 1) [17,18]. Besides, LAP also requires NADPH oxidase (nicotinamide adenine dinucleotide phosphate oxidase) activity and phagosomal reactive oxygen species (ROS) formation [18,19]. Depending on the cellular background, LAP can either accelerate or delay phagosome fusion with lysosomes [20].

To date, studies of autophagy on nonprofessional phagocytes infected with *S. aureus* provide conflicting results, with the core autophagic machinery shown to be either detrimental [21] or beneficial [22] to the infected host cells. Schnaith *et al*. have shown that *S. aureus* is taken up within RAB7-positive phagosomes of mouse embryonic fibroblasts and subsequently trapped within LC3-positive vesicles, which serve as a niche for bacterial replication. That process is dependent on bacterial virulence factors regulated by staphylococcal *agr* (accessory gene regulator). In these studies, inhibition of the core autophagy machinery by *atg5* knockout was beneficial to the host cell as it led to the reduction of intracellular *Staphylococci* [21]. It has also been shown that the staphylococcal toxin α-hemolysin, which is positively regulated by *agr*, participates in the activation of the autophagic pathway within nonprofessional phagocytes [23,24].

In contrast, Neumann *et al*. more recently demonstrated the protective role of xenophagy in *S. aureus* infection of murine fibroblasts NIH/3T3, where intracellular bacteria are ubiquitinated leading to recruitment of selective autophagy receptors such as CALCOCO2/NDP52 or SQSTM1 and *atg5* knockout leads to increased numbers of intracellular *Staphylococci* [22]. In addition, the role of the autophagic response to *S. aureus*, as beneficial or detrimental to the host, might be cell-type specific. It is also possible that the observed differences in previous studies are caused by different *S. aureus* strains used. Importantly, the autophagic response to *S. aureus* within macrophages and neutrophils has not been studied in detail, and it is currently unknown how the different processes that rely on autophagy components, such as xenophagy and LAP, are involved in the interaction of professional phagocytes with *Staphylococci* during systemic infection.

In this study, using a well-established zebrafish model of systemic staphylococcal infection [10,25] accompanied by *in vivo* imaging of transgenic zebrafish, we explore the autophagic response to *S. aureus* within professional phagocytes, such as macrophages and neutrophils. Our results identify LAP as the important pathway responsible for the staphylococcal subversion of infected neutrophils leading to the progression of systemic disease.

## Results

### S. aureus is contained within Lc3-positive vesicles in both zebrafish macrophages and neutrophils

**Figure 1.**
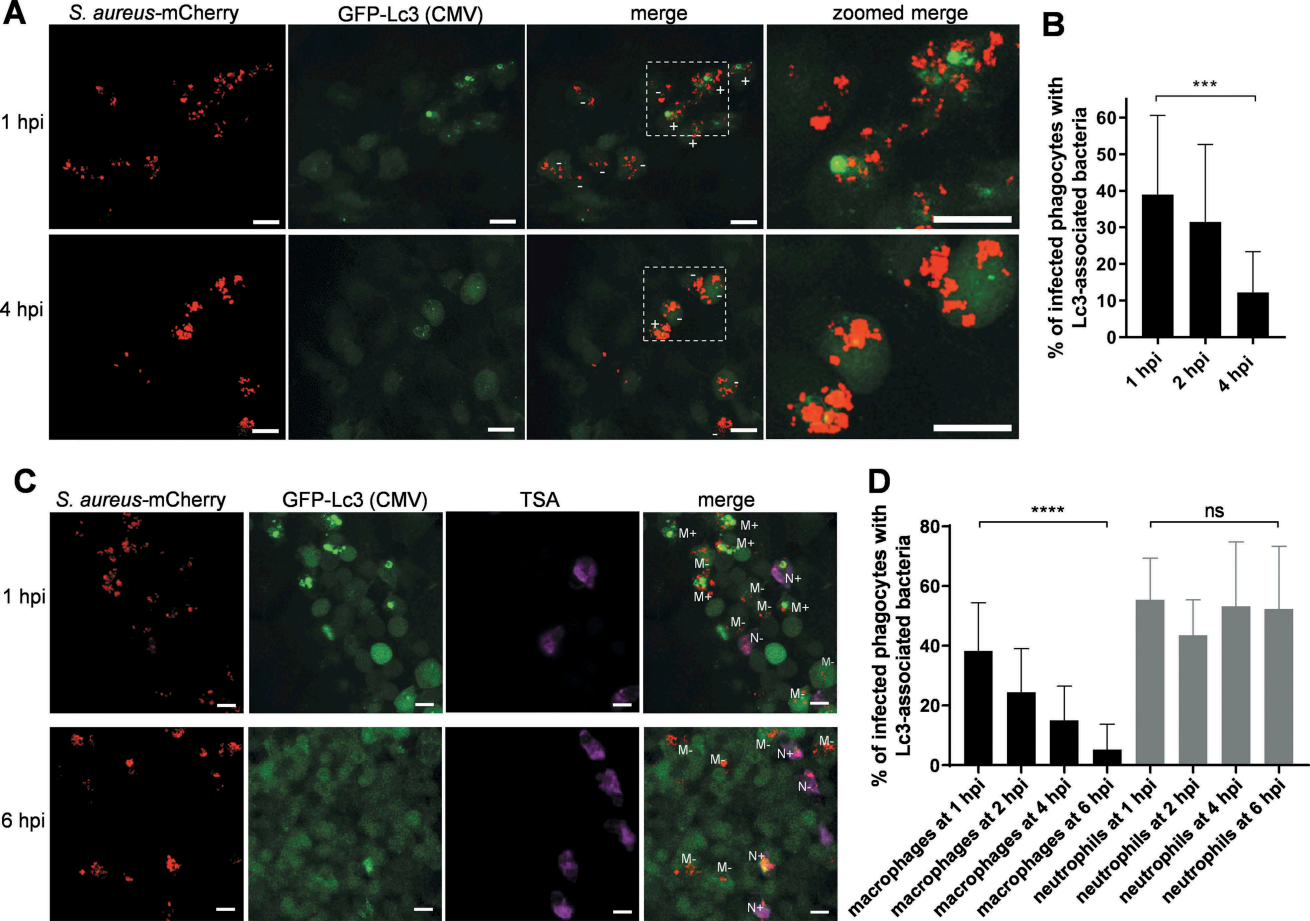
Different kinetics of the Lc3-mediated response in macrophages and neutrophils infected by *S. aureus*. (A) Confocal photomicrographs are shown as maximum intensity projections of fixed CMV:GFP-Lc3 embryos infected with approximately 1500 CFU of mCherry-labeled *S. aureus* at 1 (top panel) and 4 hpi (bottom panel). Phagocytes are seen containing bacteria with (+) or without (-) Lc3 aggregates. Images shown are representative of three independent experiments. Scale bars: 10 µm. (B) Quantification of Lc3 associations with intracellular *S. aureus* in fixed CMV:GFP-Lc3 embryos at 1, 2, and 4 hpi with approximately 1500 CFU. Data are shown as mean ± standard deviation (SD) obtained from three independent experiments (5–6 larvae per experiment per group). For 1 hpi, 175 infected phagocytes in 18 larvae were analyzed. For 2 hpi, 198 infected phagocytes in 17 larvae were analyzed. For 4 hpi, 204 infected phagocytes in 17 larvae were analyzed. One-way ANOVA with Bonferroni’s posttest was used. *** *P* < 0.001. (C) Confocal photomicrographs are shown as maximum intensity projections of fixed CMV:GFP-Lc3 transgenic embryos infected with approximately 1500 CFU of mCherry-labeled *S. aureus*. Embryos were fixed at 1 (top panel) and 6 hpi (bottom panel) and chemically stained for Mpx activity (TSA, magenta). TSA-negative macrophages are seen containing bacteria with (M+) or without (M-) Lc3 aggregates as well as TSA-positive neutrophils containing bacteria with (N+) and without Lc3 aggregates (N-). The images shown are representative of three independent experiments. Scale bars: 10 µm. (D) Quantification of Lc3 associations with intracellular *S. aureus* within macrophages (black bars) and neutrophils (gray bars) of fixed CMV:GFP-Lc3 transgenic embryos infected with approximately 1500 CFU of mCherry-labeled *S. aureus* at 1, 2, 4 and 6 hpi. Data are shown as mean ± standard deviation (SD) obtained from three independent experiments (5–6 larvae per experiment per group). For 1 hpi, 151 infected macrophages and 60 infected neutrophils in 18 larvae were analyzed. For 2, hpi 146 infected macrophages and 64 neutrophils in 18 larvae were analyzed. For 4 hpi, 159 infected macrophages and 56 neutrophils in 17 larvae were analyzed. For 6 hpi, 161 infected macrophages and 70 infected neutrophils in 18 larvae were analyzed. Two-way ANOVA with Bonferroni’s posttest was used. **** *P* < 0.0001, ns – not significant

Due to its central role in autophagy and related responses, a lipidated form of the LC3 protein has been widely used as a marker for activation of the autophagy machinery in both mammalian and zebrafish systems [26,27]. Therefore, to study the autophagic response to *S. aureus* infection at the cellular level, we used a transgenic zebrafish line *Tg(CMV:EGFP-map1lc3b)zf155* [27], hereafter called CMV:GFP-Lc3. Embryos of this line were infected with mCherry-labeled *S. aureus* SH1000 [28], as previously described [25]. We fixed the infected CMV:GFP-Lc3 zebrafish at 1, 2, and 4 h post-infection (hpi) and performed confocal microscopy to visualize the formation of Lc3-positive vesicles associated with bacteria within infected phagocytes. We observed GFP-Lc3 associations with bacteria within phagocytes (Figure 1A) with most Lc3-bacteria associations within infected phagocytes seen at 1 hpi and reducing by 4 hpi (Figure 1B).

Subsequently, to characterize the observed Lc3-mediated response specifically in infected macrophages and neutrophils, we subjected fixed CMV:GFP-Lc3 embryos to histochemical staining for endogenous peroxidase activity [25] to distinguish neutrophils from macrophages, which are peroxidase-negative in zebrafish [29]. In agreement with previous work, we observed both neutrophils and macrophages to take up *S. aureus*, with most bacteria detected within tyramide signal amplification (TSA)-negative infected macrophages [25]. We saw the number of Lc3-*S. aureus*-positive macrophages to decrease over time (Figure 1C,D), while the Lc3 associations with *S. aureus* within TSA-positive neutrophils remained high for up to 6 hpi. The Lc3 signal in infected neutrophils typically appeared in circular patterns around bacterial clusters, suggesting that it labels the vesicles containing *S. aureus*. This result demonstrates that, although both macrophages and neutrophils can mount an Lc3-mediated response to *S. aureus* infection, the kinetics of this response differs.

Infected neutrophils represent a minority of infected phagocytes in systemically infected embryonic zebrafish. Therefore, to study the neutrophil response specifically, we knocked down *irf8* (interferon regulatory factor 8), which leads to the preferential development of neutrophils at the expense of macrophages [30]. This strategy is a useful approach to manipulate neutrophil:macrophage ratios and has been successfully used in several infection studies [10,31–33]. As expected, the infection of *irf8* knockdown embryos led to a higher percentage of infected neutrophils and a higher average number of bacteria per infected neutrophil when compared to control embryos (Fig. S1A and B), which facilitated the observation of *S. aureus*-infected neutrophils. This approach also revealed that, as in the embryos with normal myeloid cell ratios, around 55% of infected neutrophils still contained Lc3-associations with bacteria at 6 hpi (Fig. S1C and D). Together, these data suggest that the processing of Lc3-positive vesicles [18] is delayed in neutrophils compared to macrophages, suggesting a potential inhibition of autophagic flux within *S. aureus*-infected neutrophils.

### A neutrophil-specific autophagy reporter line confirms the Lc3-mediated response to S. aureus in neutrophils

**Figure 2.**
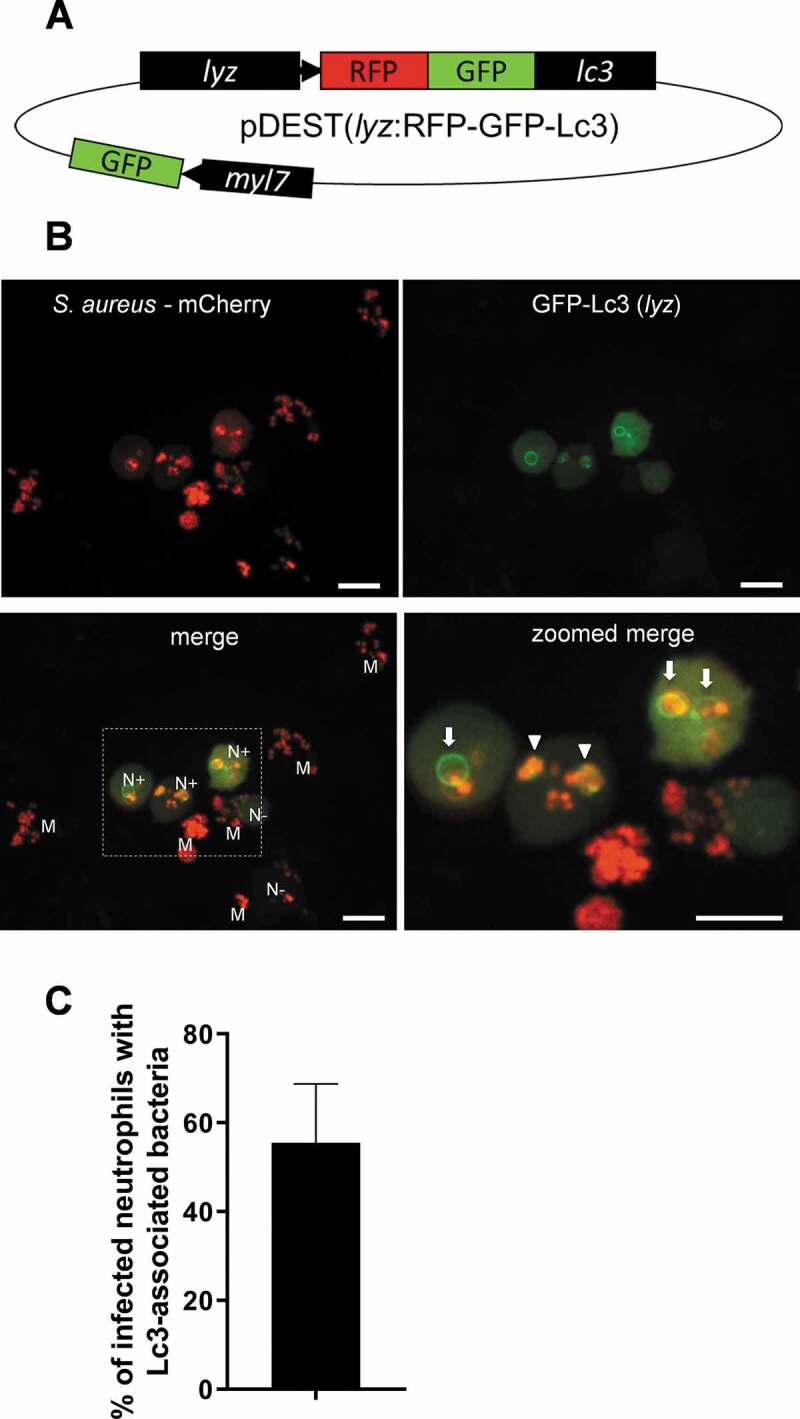
Generation of a *lyz*:RFP-GFP-Lc3 transgenic line in zebrafish confirms the Lc3-mediated response to *S. aureus* within neutrophils. (A) Schematic of the pDEST(*lyz*:RFP-GFP-Lc3) construct encoding the fusion RFP-GFP-Lc3 protein under the neutrophil-specific *lyz* promoter. In addition, the heart marker *myl7*-driven GFP is used to facilitate the screening of positive larvae. (B) Confocal images at maximum projection of the Lc3-mediated response at 1 hpi in live *lyz*:RFP-GFP-Lc3 embryos infected with approximately 1500 CFU of mCherry-labeled *S. aureus*. Lyz-positive neutrophils are seen containing bacteria with (N+) or without (N-) Lc3 aggregates. Lyz-negative macrophages are also seen containing bacteria (M). The images shown are representative of three independent experiments. Arrows indicate spacious Lc3-positive vesicles, whereas arrowheads show tightly wrapped Lc3-associated bacteria. Scale bars: 10 µm. (C) Quantification of Lc3 associations with intracellular *S. aureus* within infected neutrophils of live *lyz*:RFP-GFP-Lc3 embryos at 1 hpi with approximately 1500 CFU. Data are shown as mean ± standard deviation (SD) obtained from three independent experiments (6 larvae per experiment). 79 infected neutrophils were analyzed in 18 larvae

We speculated that macrophages could use the machinery of autophagy to aid in intracellular processing of *S. aureus* bacteria, while neutrophils are less able to do so, potentially due to the manipulation of autophagy by intracellular *Staphylococci*. To study the neutrophil-specific Lc3-mediated response without the need for additional staining, we generated a transgenic zebrafish line where zebrafish Lc3 is fused with a tandem fluorophore RFP-GFP [34] and is expressed under a neutrophil-specific promoter *lyz* [35]: *Tg(lyz:RFP-GFP-map1lc3b)sh383* transgenic line (Figure 2A), hereafter called *lyz*:RFP-GFP-Lc3.

Upon systemic infection of *lyz*:RFP-GFP-Lc3 transgenic embryos with mCherry-labeled *S. aureus*, we identified Lc3-*S.aureus* associations at 1 hpi in approximately 55% of infected *lyz*-positive neutrophils (Figure 2B,C). These bacteria-containing vesicles were both RFP- and GFP- positive, which suggests no acidification, as vesicle fusion with the acidic lysosomes quenches GFP signal. In addition, as expected, a majority of the internalized bacteria were found in *lyz*-negative (hence unlabeled) macrophages (Figure 2B). These results demonstrated that the *lyz*:RFP-GFP-Lc3 line provides a new tool for studying the Lc3-mediated response within neutrophils and allows high-quality live-imaging without the visible fluorescence of other cells, as observed in the CMV:GFP-Lc3 line (Figure 1). Live imaging of infected neutrophils in the CMV:GFP-Lc3 line showed bacteria contained within spacious rings of the Lc3 signal (Fig. S1E). In agreement, we could observe the *lyz*:RFP-GFP-Lc3 fluorescent signal encircling spacious bacteria-containing vesicles, confirming that neutrophils mount an Lc3-mediated response to *S. aureus* infection (Figure 2B).

### Functional NADPH oxidase is required for the formation of Lc3 aggregates associated with phagocytosed Staphylococci

**Figure 3.**
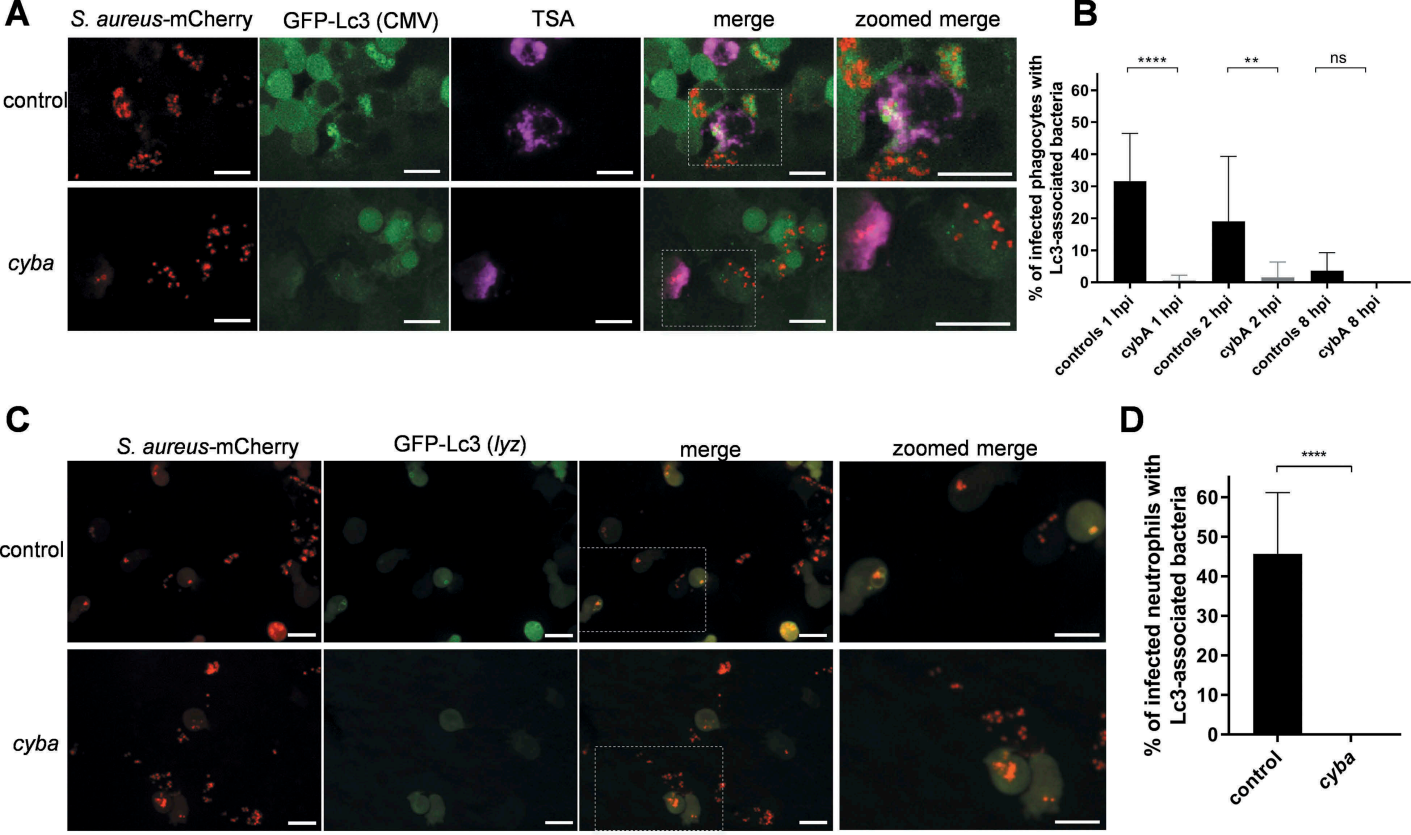
NADPH oxidase is required for the Lc3-mediated response to *S. aureus* infection. (A) Confocal photomicrographs are shown as maximum intensity projections of the Lc3-mediated response in control (top panel) and *cyba* knockdown (bottom panel) of fixed CMV:GFP-Lc3 embryos infected with approximately 1500 CFU of mCherry-labeled *S. aureus*. Embryos were fixed at 1 hpi and chemically stained for Mpx activity (TSA, magenta). Scale bars: 10 µm. (B) Quantification of Lc3 associations with intracellular *S. aureus* within infected phagocytes of fixed CMV:GFP-Lc3 control and *cyba* knockdown embryos at 1, 2, and 8 hpi with approximately 1500 CFU. Data are shown as mean ± standard deviation (SD) obtained from three independent experiments (5–6 larvae per experiment per group). For 1 hpi, 205 infected phagocytes in 18 larvae analyzed. For 2 hpi, 188 infected phagocytes in 18 larvae were analyzed. For 8 hpi, 161 infected phagocytes in 17 larvae were analyzed. Two-way ANOVA with Bonferroni’s posttest was used. **** *P* < 0.0001, ** *P* < 0.01, ns – not significant. (C) Confocal photomicrographs are shown as maximum intensity projections of the Lc3-mediated response at 1 hpi in control (top panel) and *cyba* knockdown live *lyz*:RFP-GFP-Lc3 embryos infected with mCherry-labeled *S. aureus*. Scale bars: 10 µm. (D) Quantification of Lc3 associations with intracellular *S. aureus* within infected neutrophils of live *lyz*:RFP-GFP-Lc3 embryos at 1 hpi with approximately 1500 CFU. Data are shown as mean ± standard deviation (SD) obtained from three independent experiments (6 larvae per experiment per group). 96 infected neutrophils in 18 control larvae were analyzed. 92 infected neutrophils in 18 *cyba* knockdown larvae were analyzed. Unpaired two-tailed t-test was used. **** *P* < 0.0001)

**Figure 4.**
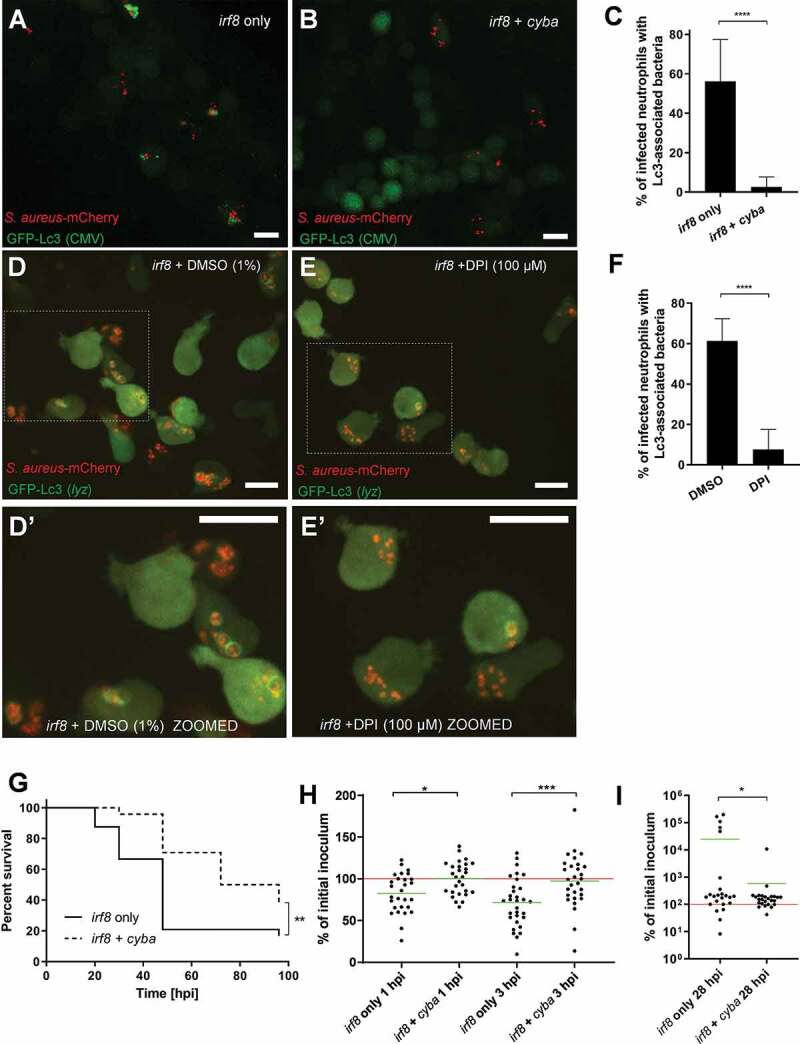
Formation of NADPH oxidase-dependent Lc3-positive vesicles containing *S. aureus* in neutrophils is detrimental for the infected host. (A and B) Confocal photomicrographs are shown as maximum intensity projections of the Lc3-mediated response at 1 hpi in *irf8*-only (A) and *irf8* + *cyba* knockdown (B) fixed CMV:GFP-Lc3 embryos infected with approximately 1500 CFU of mCherry-labeled *S. aureus*. Scale bars: 10 µm. (C) Quantification of Lc3 associations with intracellular *S. aureus* within infected neutrophils of *irf8*-only and *irf8* + *cyba* knockdown fixed CMV:GFP-Lc3 embryos at 1 hpi with approximately 1500 CFU. Data are shown as mean ± standard deviation (SD) obtained from three independent experiments (5–6 larvae per experiment per group). 174 infected neutrophils in 16 *irf8*-only knockdown larvae were analyzed. 210 infected neutrophils in 17 *irf8* + *cyba* knockdown larvae were analyzed. Unpaired two-tailed t-test was used. **** *P* < 0.0001. (D and E) Confocal photomicrographs are shown as maximum intensity projections of the Lc3-mediated response at 1 hpi in control (DMSO) (D) and DPI-treated (E) *irf8* knockdown live *lyz*:RFP-GFP-Lc3 embryos infected with approximately 1500 CFU of mCherry-labeled *S. aureus*. The images shown are representative of three independent experiments. Scale bars: 10 µm. (**D’ and E’**). Zoomed-in fragments of photomicrographs d and e. (F) Quantification of Lc3 associations with intracellular *S. aureus* within infected neutrophils of control (DMSO) and DPI-treated *irf8* knockdown live *lyz*:RFP-GFP-Lc3 embryos at 1 hpi with approximately 1500 CFU. Data are shown as mean ± standard deviation (SD) obtained from three independent experiments. 252 infected neutrophils in 18 DMSO-treated larvae were analyzed. 208 infected neutrophils in 17 DPI-treated larvae were analyzed. Unpaired two-tailed t-test was used.**** *P* < 0.0001. (G) Survival of *irf8-*only or *irf8* + *cyba* knockdown zebrafish larvae following intravenous injection with approximately 1500 CFU of *S. aureus* at 30 hpf (25 larvae per group). This result is representative of three independent experiments. Survival curves were compared using a log-rank (Mantel-Cox) statistical test. ** *P* < 0.01. (H and I) The CFU counts of the *irf8-*only or *irf8* + *cyba* knockdown larvae infected intravenously with approximately 1500 CFU of *S. aureus* at 1 and 3 hpi (H) or 28 hpi (I). At each timepoint, larvae were sacrificed, homogenized, and the recovered staphylococci were enumerated by serial dilutions. The red line represents the level of the initial inoculum, whereas the green lines represent the mean value of each group. Data are obtained from 3 independent experiments (n of larvae per timepoint ≥24). One-way ANOVA with Bonferroni’s posttest was used for (**H**) and an unpaired two-tailed t-test was used for (**I**). * *P* < 0.05, *** *P* < 0.001

We have shown that that the transgenic Lc3 markers used in this study label bacteria-containing vesicles, suggesting these are formed by either selective autophagy or Lc3-associated phagocytosis (LAP). To distinguish these possibilities, we manipulated phagosomal ROS production specifically required for LAP [18,19,33,36]. Using well-validated morpholino-modified antisense oligonucleotide injection [37], we knocked down the expression of *cyba/p22phox* (cytochrome b-245 alpha chain), a membrane-bound subunit of phagocyte NADPH oxidase. Loss of Cyba led to a near-complete absence of the Lc3-*S.aureus* association in both macrophages and neutrophils of CMV:GFP-Lc3 embryos (Figure 3A,B). In agreement, infection of *cyba* knockdown *lyz*:RFP-GFP-Lc3 embryos led to no Lc3-*S. aureus* associations (Figure 3C,D). In addition, Lc3-bacteria associations were lost upon genetic (Figure 4A–C) and diphenyleneiodonium (DPI)-mediated chemical inhibition of NADPH oxidase (Figure 4D,F) in macrophage-depleted, neutrophil-enriched (*irf8* knockdown) larvae. Therefore, these results suggest that Cyba and hence the NADPH oxidase complex plays an important role in the recruitment of Lc3 to *S. aureus* in both macrophages and neutrophils, and therefore, we propose that this response represents LAP.

To further determine what effect the inhibition of NADPH oxidase activity and the associated Lc3 response have on the staphylococcal pathogenesis in the neutrophil-enriched (*irf8* knockdown) zebrafish, we performed a survival experiment with *S. aureus*-infected embryos. Strikingly, the survival of infected zebrafish with genetically or pharmacologically inhibited NADPH oxidase was higher than controls (Figure 4G and S2A), suggesting that the formation of NADPH oxidase-mediated Lc3-positive vesicles containing *S. aureus* in neutrophils is detrimental for the infected host. Importantly, the percentage of infected neutrophils, as well as the average number of bacteria per infected neutrophil, remained the same in control and treated groups (Fig. S2B-E). The observed difference in host survival prompted us to enumerate bacteria within larvae following infection. We found that within the first 3 h of infection, the neutrophils of *cyba/irf8* double knockdown embryos are significantly less proficient in killing the internalized bacteria than the *irf8*-only knockdown embryos (Figure 4H). However, in line with the survival curves (Figure 4G), at the later time point (28 hpi), higher numbers of *Staphylococci* were found in a subset of the *irf8-*only knockdown embryos compared with the *cyba/irf8* double knockdown embryos (Figure 4I). Therefore, the early reduction in the killing of internalized *S. aureus* by *cyba*-deficient embryos ultimately resulted in the increased host survival and less *in vivo* bacteria in infected embryos at 28 hpi. To corroborate the results obtained with the *irf8* knockdown, we used a second strategy to explore the impact of macrophage ablation (clodronate-containing liposomes) to deplete macrophages while not affecting neutrophils, especially [38]. Similar to *irf8* knockdown, the *cyba*-deficient, macrophage-depleted larvae were more resistant to *S. aureus* than their respective controls (Fig. S2F). Interestingly, the loss of *cyba* had no effect on the survival of infected larvae in the presence of macrophages (Fig. S2G), suggesting that NADPH oxidase-mediated processing of *S. aureus* in zebrafish macrophages does not play a vital role in intracellular handling of bacteria. We conclude that the formation of NADPH oxidase-mediated, Lc3-positive vesicles containing *S. aureus* in neutrophils is detrimental for the infected host.

Since the NAPDH oxidase can also influence *S. aureus* in a LAP-independent manner, including both cell-intrinsic and extrinsic mechanisms, we decided to target LAP in a way that does not affect the NADPH complex. In order to determine whether the elevated resistance to *S. aureus* of *cyba* knockdown-mediated neutrophil-enriched zebrafish larvae is due to loss of LAP or the NAPDH oxidase itself, we targeted the LAP machinery by simultaneously knocking down *atg5* and *atg16l1* (autophagy related 16 like 1) without affecting the NADPH oxidase complex.

As expected, the *atg5* + *atg16l1* double knockdown led to a significant decrease (near-complete loss) of Lc3-bacteria associations within *S. aureus*-infected neutrophils of *irf8* knockdown zebrafish larvae (Fig. S3A-C). In addition, the *atg5* + *atg16l1* double knockdown embryos were significantly more resistant to *S. aureus* infection (Fig. S3D), phenocopying the effect seen where LAP was inhibited via *cyba* knockdown. This result further confirms that the loss of LAP leads to elevated resistance to *S. aureus* infection, and LAPosomes containing *Staphylococci* could serve as an intracellular niche facilitating disease progression.

### NADPH oxidase-dependent neutrophil response to S. aureus forms spacious non-acidified Lc3-positive phagosomes

**Figure 5.**
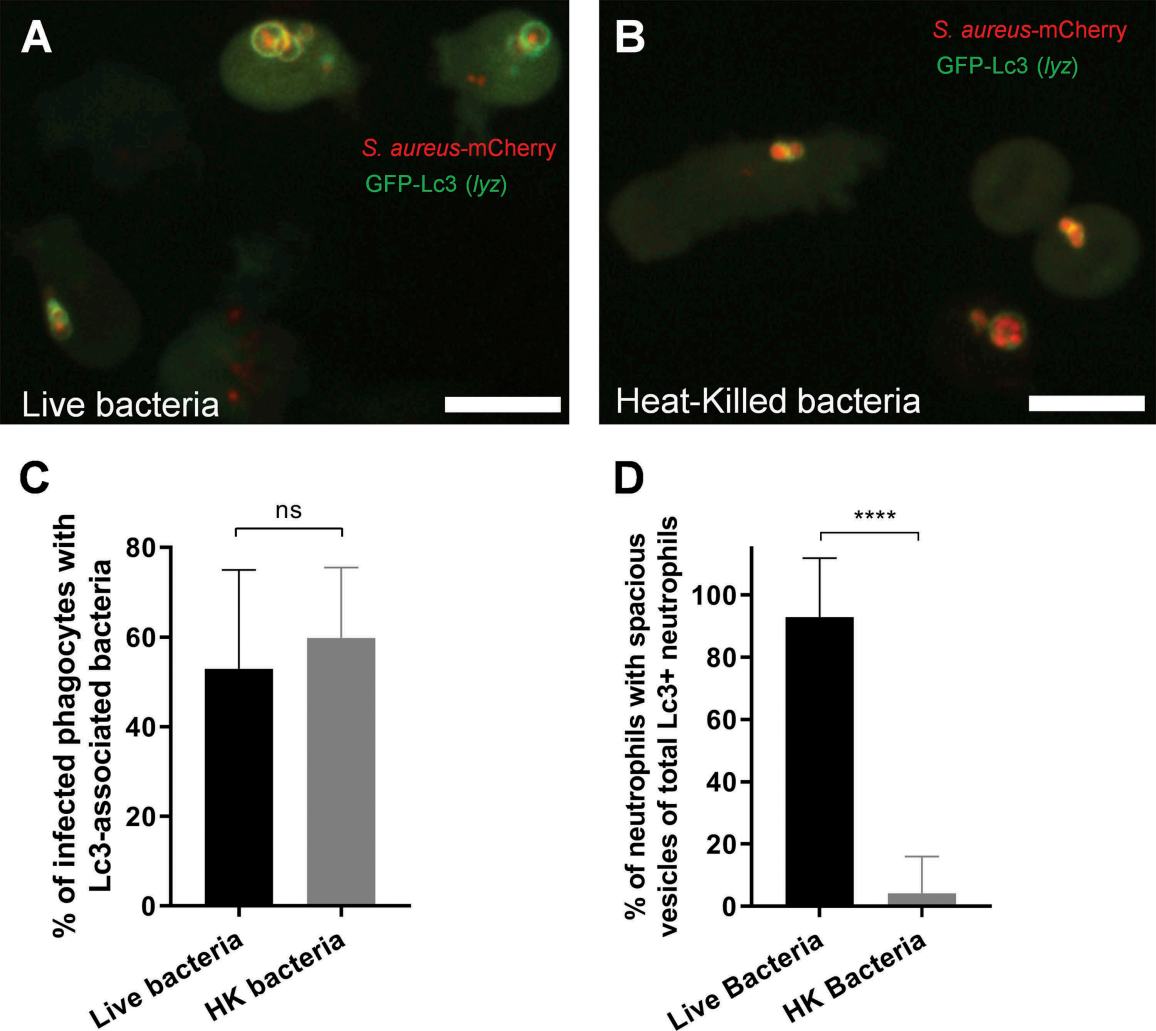
The Lc3-mediated response in neutrophils occurs to both live and heat-killed *S. aureus*, but spacious Lc3-positive vesicles are formed only with live bacteria. (A and B) Confocal photomicrographs are shown as maximum intensity projections of the Lc3-mediated response at 1 hpi in live *lyz*:RFP-GFP-Lc3 embryos infected with approximately 1500 CFU of live (A) or heat-killed (B) mCherry-labeled *S. aureus*. Scale bars: 10 µm. (C) Quantification of Lc3 associations with intracellular *S. aureus* at 1 hpi within neutrophils of live *lyz*:RFP-GFP-Lc3 embryos infected with approximately 1500 CFU of live or heat-killed (HK) mCherry-labeled *S. aureus*. Data are shown as mean ± standard deviation (SD) obtained from three independent experiments. 80 infected neutrophils in 16 larvae injected with live bacteria were analyzed. 72 infected neutrophils in 16 larvae injected with heat-killed bacteria were analyzed. Unpaired two-tailed t-test was used. ns – not significant. (D) Quantification of neutrophils with spacious *S. aureus*-containing phagosomes at 1 hpi within live *lyz*:RFP-GFP-Lc3 embryos infected with approximately 1500 CFU of live or heat-killed (HK) mCherry-labeled *S. aureus*. Data are shown as mean ± standard deviation (SD) obtained from three independent experiments. 46 infected Lc3-positive neutrophils in 16 larvae injected with live bacteria were analyzed. 44 infected neutrophils Lc3-positive in 16 larvae injected with heat-killed bacteria were analyzed. Unpaired two-tailed t-test was used. **** *P* < 0.0001

Since we saw Lc3 persist on phagosomes in infected neutrophils, we decided to characterize this potentially host-detrimental response further and assess the pH status of internalized bacteria. In order to determine whether live bacteria are required to induce an Lc3-mediated response within neutrophils, we injected heat-killed *Staphylococci* into *lyz*:RFP-GFP-Lc3 embryos. This method resulted in similar levels of Lc3-bacteria associations within infected neutrophils when compared to neutrophils infected with live bacteria (Figure 5A–C) suggesting that the observed Lc3-mediated response does not require bacterial-driven damage of the phagosome or subsequent bacterial escape into the cytoplasm, similar to what has been recently proposed for LC3 recruitment to *Listeria monocytogenes* [39]. However, only live bacteria led to the formation of spacious Lc3-positive phagosomes within infected neutrophils by 1 hpi (Figure 5D and S3E). Therefore, these characteristic spacious phagosomes in *S. aureus*-infected neutrophils may be indicative of bacterial pathogenesis.

**Figure 6.**
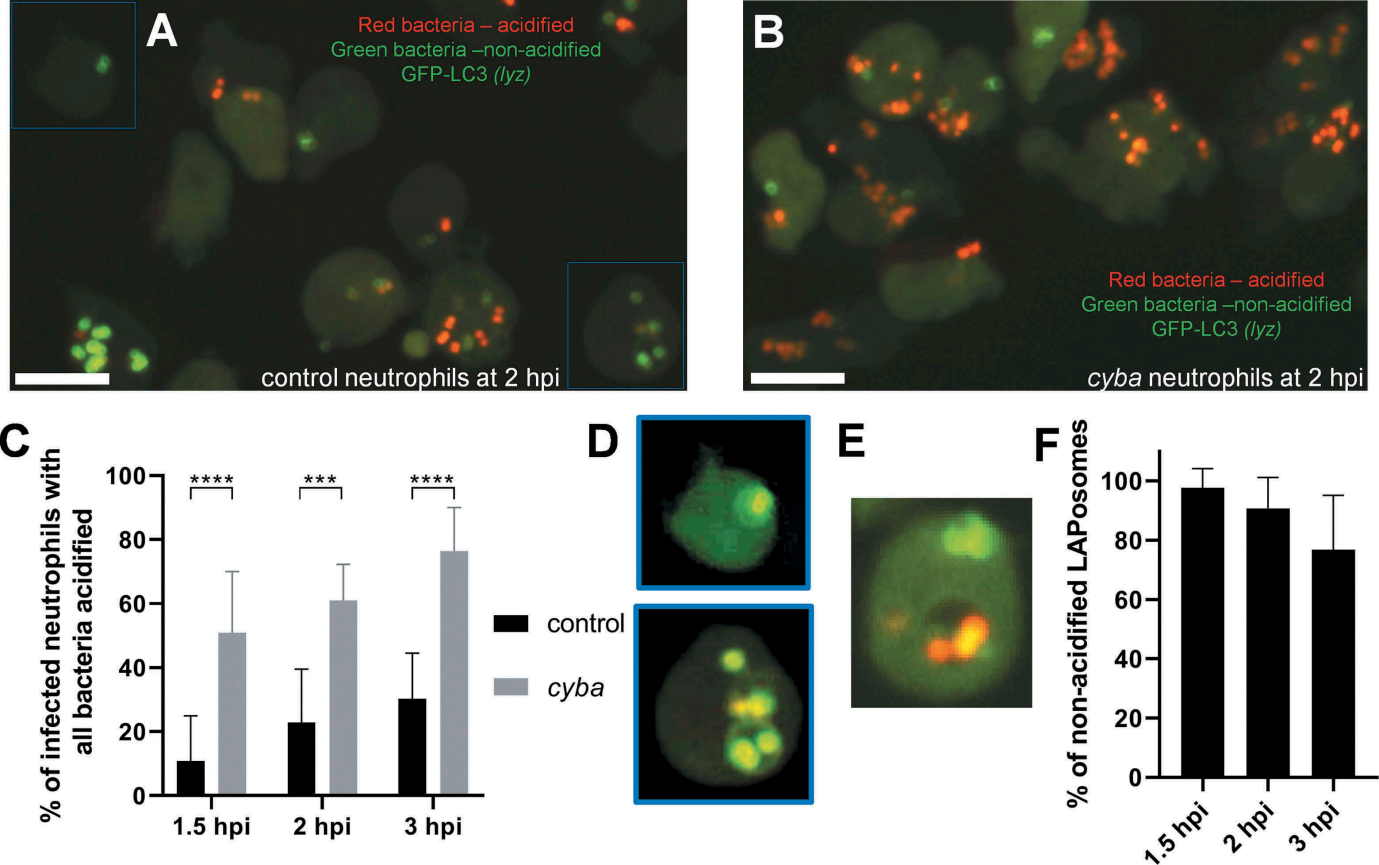
Loss of Cyba leads to increased acidification of neutrophil-ingested *S. aureus*. (A and B) Confocal photomicrographs shown as maximum intensity projections of the control (A) and *cyba* knockdown (B) live *lyz*:RFP-GFP-Lc3 embryos infected with approximately 1500 CFU of *S. aureus* stained with pHrodo Red and Fluorescein pH-indicating dyes. Green bacteria indicate that they are localized in neutral pH, whereas red bacteria are acidified. Scale bars: 10 µm. (C) Quantification of acidification rates at 1.5, 2, and 3 hpi of intracellular *S. aureus* within control and *cyba* knockdown neutrophils of live *lyz*:RFP-GFP-Lc3 embryos infected with approximately 1500 CFU of *S. aureus*. Data are shown as mean ± standard (SD) obtained from three independent experiments (6 larvae per group). For 1.5 hpi, 170 infected neutrophils in 18 control larvae, and 168 infected neutrophils in 18 larvae were analyzed. For 2 hpi, 188 infected neutrophils in 18 control larvae and 214 infected neutrophils in 18 *cyba* larvae were analyzed. For 3 hpi, 154 infected neutrophils in 18 control larvae and 204 neutrophils in 18 cyba larvae were analyzed. Two-way ANOVA with Bonferroni’s posttest was used. *** P < 0.001, **** P < 0.0001. (D) Examples of control neutrophils (indicated in panel a) with LAPosomes containing non-acidified *S. aureus at* 2 hpi. Contrast was enhanced equally for both channels to visualize LAPosomes. (E) A rare example of a control neutrophil with a LAPosome containing acidified *S. aureus* at 3 hpi. (F) Quantification of non-acidified LAPosomes within control neutrophils of live *lyz*:RFP-GFP-Lc3 embryos at 1.5, 2 and 3 hpi. For 1.5 hpi, 142 of LAPosomes were analyzed. For 2 hpi, 157 of LAPosomes were analyzed. For 3 hpi, 132 of LAPosomes were analyzed

To assess whether ingested *S. aureus* was in acidic compartments, they were stained prior to inoculation with a combination of pH-sensitive dyes – pHrodo red and fluorescein succinimidyl esters (bacteria are green in neutral pH and red in acidic pH). While bacteria in control neutrophils were not in acidified compartments early during infection, the proportion of bacteria in acidified compartments increased slightly at later stages of infection (Figure 6A,C). In *cyba* knockdown neutrophils, significantly more bacteria localized in acidic compartments (Figure 6B,C). Importantly, a vast majority of spacious Lc3-positive compartments containing bacteria in control fish remained at neutral pH (Figure 6D–F). Therefore, a lack of acidification of such phagosomes can potentially provide a non-acidified intraphagocyte niche for bacterial persistence or replication.

### The selective autophagy receptor Sqstm1/p62 is recruited to Staphylococci ingested by neutrophils

**Figure 7.**
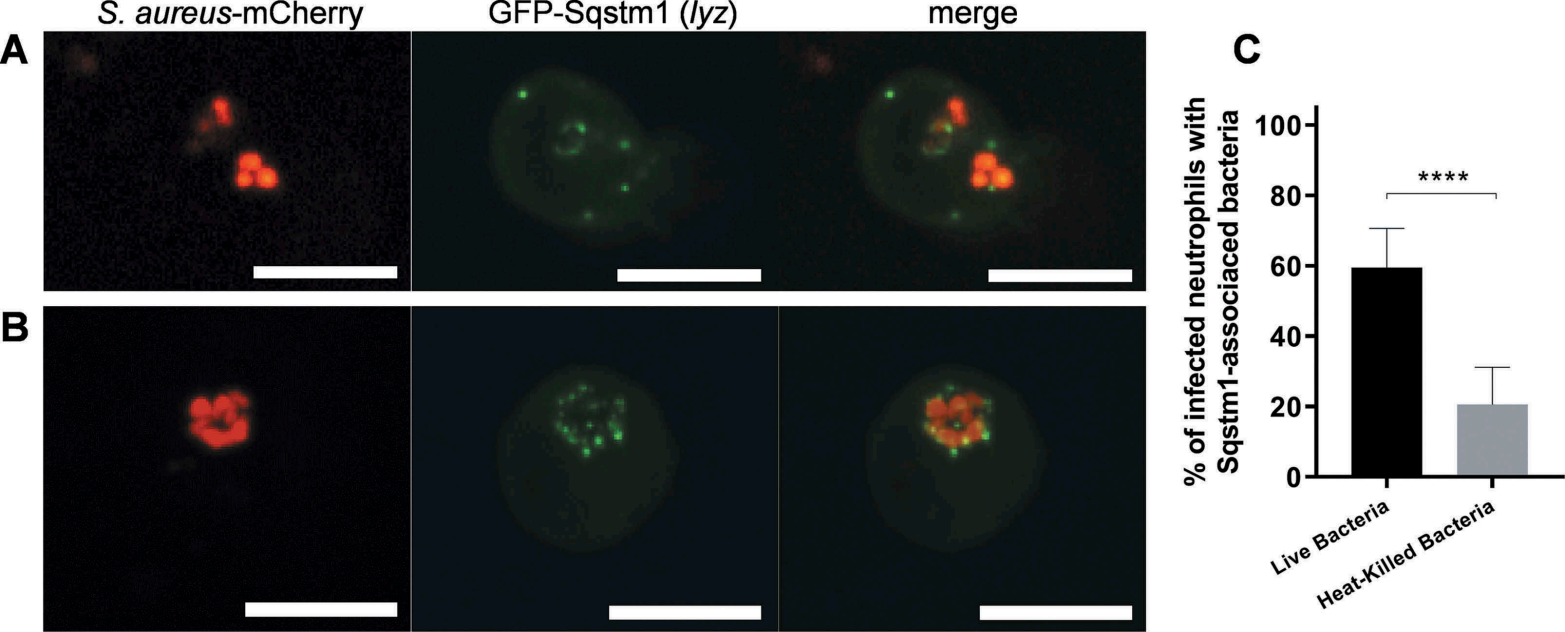
*S. aureus* within neutrophils is targeted by selective autophagy receptor Sqstm1. (A and B) Confocal photomicrographs of Sqstm1-mediated response at 1 hpi in live *lyz*:GFP-Sqstm1 embryos infected with mCherry-labeled *S. aureus*. The fusion GFP-Sqstm1 protein colocalizes with intracellular bacteria (A) or with apparent vesicles containing the bacteria (B). The images shown are representative of three independent experiments. Scale bars: 10 µm. (C) Quantification of Sqstm1 associations with intracellular *S. aureus* at 2 hpi within neutrophils of live *lyz*:GFP-Sqstm1 embryos infected with approximately 1500 CFU of live or heat-killed mCherry-labeled *S. aureus*. Data are shown as mean ± standard deviation (SD) obtained from three independent experiments (5–6 larvae per group per experiment). 109 infected neutrophils in 18 larvae injected with live bacteria were analyzed. 101 infected neutrophils in 17 larvae injected with heat-killed bacteria were analyzed. Unpaired two-tailed t-test was used. **** P < 0.0001

Selective autophagy is involved in *S. aureus* infection of nonprofessional phagocytes [22], where evidence of bacterial ubiquitination and SQSTM1/p62 recruitment was demonstrated. To confirm whether *S. aureus* in neutrophils recruits Sqstm1, we used a *Tg(lyz:GFP-sqstm1/p62)i330* line [40] hereafter called *lyz*:GFP-Sqstm1, in which we fused the autophagy receptor protein Sqstm1 to GFP under the neutrophil-specific *lyz* promoter. We infected the *lyz*:GFP-Sqstm1 larvae with mCherry-expressing *S. aureus* and subjected to spinning confocal disk imaging. Within *S. aureus*-infected neutrophils, Sqstm1 commonly colocalizes with intracellular bacteria (Figure 7A), also with apparent bacteria-containing vesicles (Figure 7B). We observed Sqstm1-*S. aureus* colocalization in neutrophils in approximately 60% of infected neutrophils (Figure 7C), although the pattern of Sqstm1 decoration differed from Lc3 (Figure 2B). We observed GFP-Sqstm1 puncta surrounding the bacteria or possibly vesicles containing bacteria in neutrophils rather than whole (often spacious) Lc3-positive vesicles. In contrast to Lc3 (Figure 5), we observed significantly less neutrophil Sqstm1-*S. aureus* associations when we used heat-killed bacteria (Figure 7C) suggesting that live bacteria may be required to damage and/or escape the phagosomes and recruit xenophagy receptors such as Sqstm1. Therefore, these results suggest that xenophagy might also occur within *S. aureus*-infected neutrophils, and this effect is downstream of the initial recruitment of Lc3 to phagosomes.

**Figure 8.**
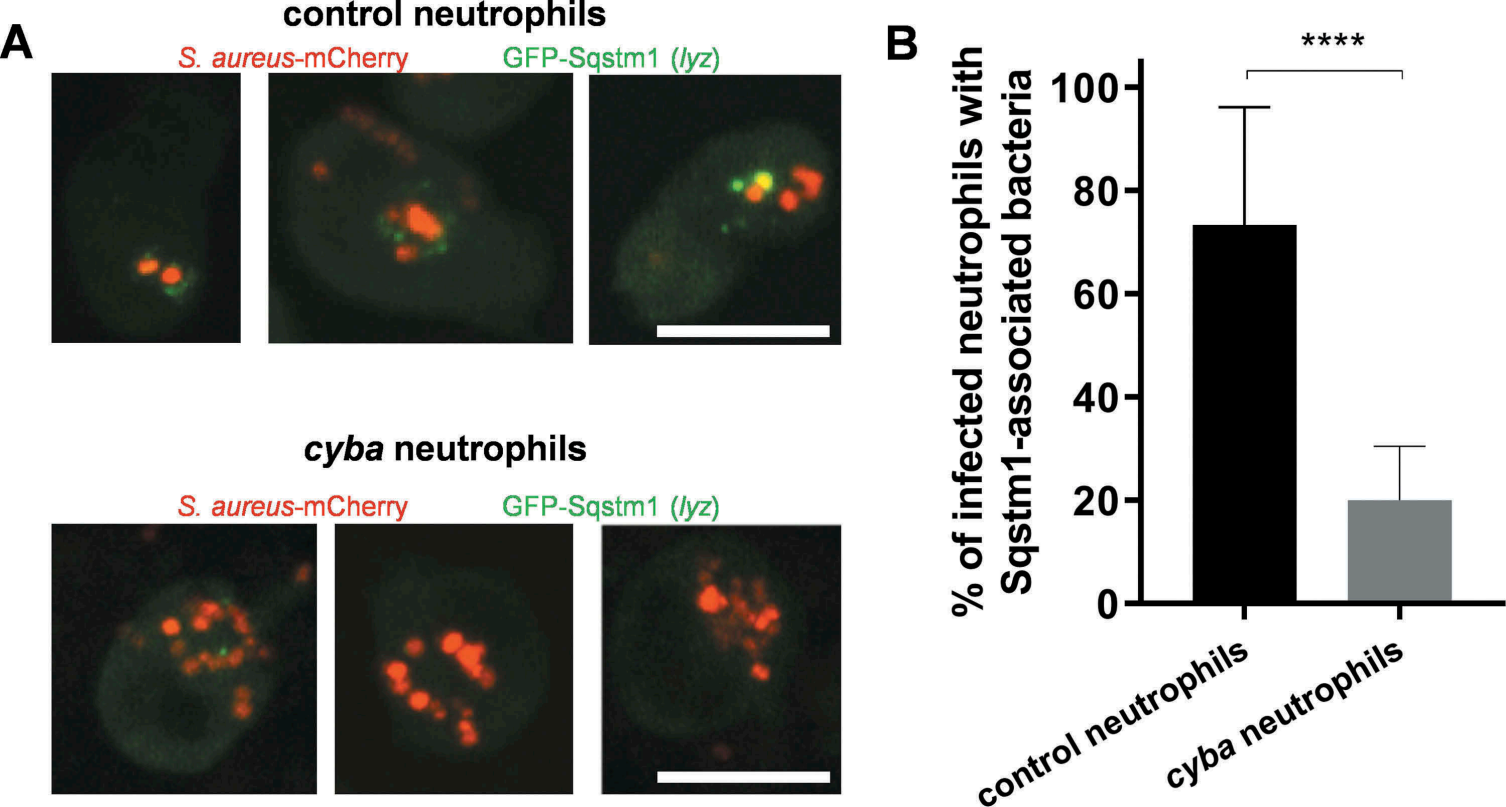
Diminished Sqstm1 recruitment to *S. aureus* in LAP-deficient neutrophils. (A) Confocal photomicrographs of Sqstm1-mediated response at 2 hpi in live control (top panel) and *cyba* knockdown (bottom panel) *lyz*:GFP-Sqstm1 embryos infected with mCherry-labeled *S. aureus*. The images shown are representative of three independent experiments. Scale bars: approximately 10 µm. (B) Quantification of Sqstm1 associations with intracellular *S. aureus* at 2 hpi within neutrophils of live *lyz*:GFP-Sqstm1 infected with mCherry-labeled *S. aureus*. Data are shown as mean ± standard deviation (SD) obtained from three independent experiments. 66 infected neutrophils in 17 control larvae were analyzed. 90 infected neutrophils in 19 *cyba* knockdown larvae were analyzed. Unpaired two-tailed t-test was used. **** P < 0.0001

We also hypothesized that if Lc3-associated phagosomes (LAPosomes) formed within infected neutrophils damaged by *Staphylococci*, suppression of LAP would lead to the reduction of Sqstm1/p62 associations with intracellular bacteria. In order to inhibit LAP, we performed a *cyba* knockdown and quantified the formation of Sqstm1-positive structures associated with intracellular bacteria. Indeed, we observed significantly less Sqstm1 association with intracellular bacteria in neutrophils, when LAP was blocked by *cyba* knockdown, suggesting that Sqstm1 association with bacteria is downstream of LAP (Figure 8). Approximately 20% of neutrophils remained Sqstm1-associated in the absence of *cyba* (Figure 8B), while the association with Lc3-bacteria was abolished almost completely (Figure 3B). This lack of Lc3 recruitment suggests that the remaining Sqstm1-associated bacteria in LAP-deficient embryos may not be targeted to selective autophagy.

### Lc3 recruitment to S. aureus phagosomes is independent of the selective autophagy receptor Sqstm1

**Figure 9.**
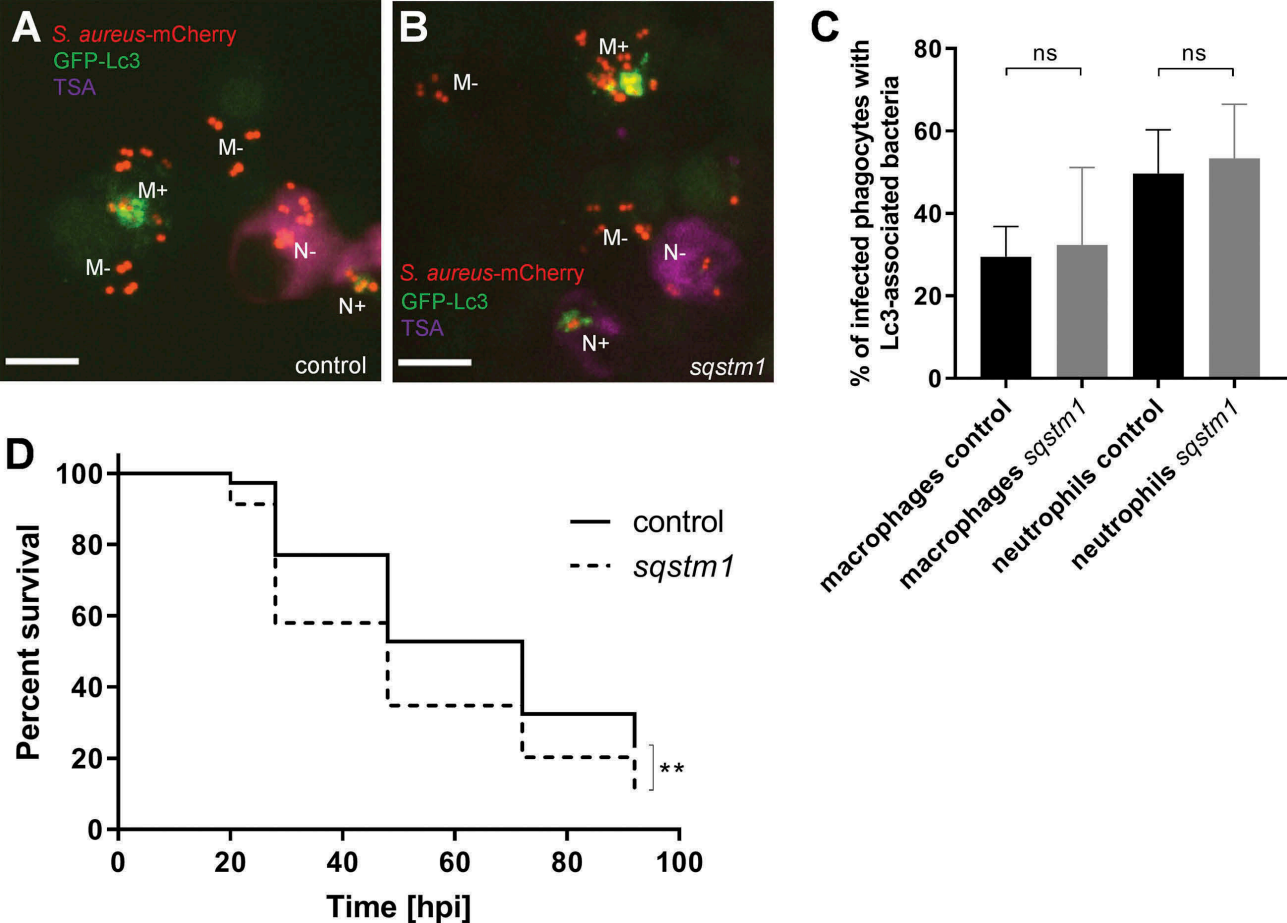
Loss of Sqstm1 leads to increased susceptibility to *S. aureus* infection. (A and B) Confocal photomicrographs are shown as maximum intensity projections of the Lc3-mediated response at 1 hpi within infected macrophages and neutrophils of control (A) and *sqstm1* knockdown (B) fixed CMV:GFP-Lc3 embryos infected with mCherry-labeled *S. aureus*. Embryos were fixed at 1 hpi and chemically stained for Mpx activity (TSA, magenta). TSA-negative macrophages are seen containing bacteria with (M+) or without (M-) Lc3 aggregates. TSA-positive neutrophils contain bacteria with (N+) and without Lc3 aggregates (N-). The images shown are representative of three independent experiments. Scale bars:10 µm. (C) Quantification of Lc3 associations with intracellular *S. aureus* at 1 hpi within infected macrophages and neutrophils of control and *sqstm1* knockdown fixed CMV:GFP-Lc3 embryos. Data are shown as mean ± standard deviation (SD) obtained from three independent experiments. 174 infected macrophages and 72 neutrophils were analyzed in 18 control larvae. 165 infected macrophages and 68 neutrophils were analyzed in *sqstm1* knockdown larvae. One-way ANOVA with Bonferroni’s posttest was used. ns – not significant. (D) Survival of *irf8-*only or *irf8* + *sqstm1* knockdown zebrafish larvae following intravenous injection with S. aureus at 30 hpf (≥69 larvae per group). This result is obtained from three independent experiments. Survival curves were compared using a log-rank (Mantel-Cox) statistical test. ** *P* < 0.01

Neumann *et al*. have previously proposed that anti-staphylococcal SQSTM1-dependent xenophagy is protective of nonprofessional phagocytes infected by *S. aureus*, although to a limited extent [22]. Similarly, our recent work in zebrafish has revealed that Sqstm1 is beneficial to zebrafish larvae infected by *S. aureus* [40]. To extend our understanding of the role of Sqstm1 in *S. aureus*-infected macrophages and neutrophils, we studied the effect of Sqstm1 deficiency on Lc3 recruitment to *S. aureus* in macrophages and neutrophils. We confirmed the efficacy of knockdown using an *sqstm1* splice morpholino [41] by RT-PCR (Fig. S4A), and we subsequently infected zebrafish embryos with *S. aureus*. We found that the formation of Lc3-positive phagosomes in both macrophages and neutrophils was *sqstm1*-independent as morpholino-mediated *sqstm1* knockdown did not lead to the reduction of Lc3-*S.aureus* association (Figure 9A–C), further supporting that the initial Lc3-mediated response is indeed LAP and is *sqstm1*-independent. However, in neutrophil enriched, macrophage-depleted larvae, loss of *sqstm1* caused a mild but statistically significant increase in susceptibility to *S. aureus* suggesting that *sqstm1*-mediated processes, perhaps occurring following damage to the LAPosomes, are protective for *S. aureus*-infected neutrophils (Figure 9D). Importantly, the percentage of infected neutrophils, as well as the average number of bacteria per infected neutrophil, remained the same in control and treated groups (Fig. S4B and C). Together, we propose that *S. aureus* exploits the autophagic response in neutrophils to establish an intracellular niche in LAPosomes, while an *sqstm1*-dependent mechanism in neutrophils may counteract the intracellular growth of the pathogen at later stages of infection.

## Discussion

*Staphylococcus aureus* has been shown to elicit an autophagic response in nonprofessional phagocytes as well as dendritic cells and macrophages [21,22,42,43], while autophagic responses to *S. aureus* in neutrophils have not been studied to date. To be able to utilize the autophagic machinery as a potential therapeutic target, several aspects of this host response need to be determined. First, what is the nature of the autophagic process targeting *S. aureus*, i.e., xenophagy or LAP? Secondly, what are the functional consequences of the autophagic response on different types of infected host cells? Do these processes promote bacterial pathogenesis or clearance within professional phagocytes infected with *S. aureus*? Using an established model of *S. aureus* infection in larval zebrafish, we demonstrated in this study that the autophagic machinery in neutrophils contributes to staphylococcal pathogenesis and that inhibition of this response improves host resistance.

Our findings relating to neutrophil function in the context of a whole organism are consistent with previous reports performed on *in vitro* cultured nonprofessional phagocytes [21,23], wherein the autophagic machinery of host cells provided a niche for staphylococcal dissemination. We propose that, at the early stages of *S. aureus* infection, infected phagocytes undergo Lc3-associated phagocytosis. This observation is because this Lc3-mediated response occurs rapidly post-infection (within 1 hpi), the Lc3 signal labels the membrane of spacious phagosomal compartments, Lc3 recruitment does not require live *Staphylococci*, and Lc3 recruitment is independent of Sqstm1, suggesting damage to the phagosomal membrane is not required. Moreover, the formation of *S. aureus*-containing Lc3-positive phagosomes required ROS production by phagocyte NADPH oxidase, another hallmark of LAP [18,19,44].

In addition to demonstrating the NADPH oxidase-dependent recruitment of Lc3 in *S. aureus* infected neutrophils, we observed that the Lc3 association with *S. aureus*-containing phagosomes in neutrophils became prolonged up to at least 6 hpi in comparison to the response observed in macrophages. Huang *et al*. observed similar results where neutrophils treated with IgG-coated beads also showed LC3 associations with phagosomes in a DPI-sensitive manner for extended periods [44]. Therefore, it seems that the recognition and subsequent clearance of LC3-labeled phagosomes through the autophagic pathway can be strongly inhibited in neutrophils, which can be utilized by intracellular microbes for pathogenesis. Using a novel zebrafish transgenic line *lyz*:RFP-GFP-Lc3 to study the Lc3 association specifically in neutrophils, we observed the formation of spacious Lc3-positive vesicles. Although observed in a different phagocyte type, these are similar to previously reported spacious Listeria-containing phagosomes (SLAPs) of mouse macrophages, the LC3-positive structures associated with persistent disease [19,45]. Our live imaging studies in zebrafish are consistent with electron microscopy data of murine neutrophils, where virulent *Staphylococci*, but not an attenuated *sar* mutant strain, also induced the formation of spacious phagosomes [8]. In another study performed on murine bone marrow-derived dendritic cells, it was demonstrated that *S. aureus* could inhibit autophagic flux, and chemical inhibition of the autophagic response by 3-MA reduced cytotoxicity caused by phagocytosed *Staphylococci* [46], resembling the response observed in neutrophils in our study.

Importantly, we showed a pronounced difference in Lc3-mediated response between macrophages and neutrophils, where we observed more neutrophils with *Staphylococci*-containing LAPosomes than macrophages, and this difference was especially more apparent at later stages of infection. Therefore, it is likely that LAP also occurs in zebrafish macrophages, but this response might be quickly resolved to lead to the subsequent loss of Lc3 association with the phagosome upon fusion with lysosomes. Indeed, using a *Salmonella* infection model, we have recently shown that zebrafish macrophages can mount a LAP response that promotes bacterial clearance [33]. Similar to our results, a study using murine RAW264.7 macrophages infected by *S. aureus* showed that the Lc3-mediated response peaks at 1 hpi and subsequently drops until 4 hpi [43]. Another recent work on RAW264.7 macrophages revealed that only a fraction of phagosomes containing *S. aureus* were Lc3-positive within 12 h of infection, indicating a low level of autophagic response and little phagosomal damage caused by *Staphylococci* within infected macrophages [47]. Thus, it appears that LAP might also occur in infected macrophages, but unlike in neutrophils, this response is rapidly processed by the autophagic flux, and hence a time-dependent loss of Lc3-bacteria associations is generally observed.

Chronic granulomatous disease (CGD) patients are more susceptible to *S. aureus* infection, predominantly manifested by liver abscesses and skin and soft tissue infections [48] but not septicemia. The exact reason why CGD patients are more prone to these staphylococcal infections is not fully understood. Although neutrophils are considered major ROS-producing phagocytes, it has been recently shown that the effect of NADPH oxidase inhibition is more pronounced in macrophages as *Ncf1*/*p47phox* (neutrophil cytosolic factor 1) mutant mice with ectopic expression of *Ncf1* in monocytes/macrophages are protected from experimental *S. aureus* systemic infection [49]. Therefore, the exact role of NADPH oxidase in neutrophils needs to be evaluated in the context of both local and systemic staphylococcal infection. In addition, our results are in line with a recent study which has shown that human neutrophils devoid of phagosomal ROS production due to NADPH oxidase mutation, although not being able to kill intracellular bacteria, are fully capable of containing *Staphylococci* in a 3D matrix, *in vitro* model. Moreover, a subset of human neutrophils with the highest acidification rate is the most efficient in containing the staphylococcal infection [50]. Perhaps this enhanced ability to contain *Staphylococci* is host-protective in our bacteremia model of infection, and the host-detrimental effect of LAP in *S. aureus*-infected zebrafish is likely due to the formation of a non-acidified niche driven by live *S. aureus*, which could subsequently lead to neutrophil lysis and bacterial escape.

Recently, Sqstm1 has been shown to play a protective role in *S. aureus* infection of zebrafish larvae, where most of the bacteria are handled by macrophages [40]. Here, in our neutrophil-enriched and macrophage-depleted model, we observed that Sqstm1 also targets *S. aureus* within infected zebrafish neutrophils and loss of Sqstm1 led to mildly increased susceptibility, indicating that Sqstm1-mediated mechanism plays a protective role in *S. aureus* infection of neutrophils and xenophagy might be involved. The observed modest effect of *sqstm1* knockdown could be due to redundancies with other autophagy receptors such as CALCOCO2/NDP52 or OPTN/optineurin [22]. Similarly, an *in vitro* work by Neumann *et al*. reports that SQSTM1 decorates and encapsulates *S. aureus* within autophagosomes of nonprofessional phagocytes and that blocking autophagosome formation by *atg5* knockout leads to higher bacterial loads [22]. On the other hand, we have observed that blocking LAP by *cyba* knockdown leads to reduced Sqstm1 recruitment to bacteria in neutrophils, suggesting that formed LAPosomes subsequently undergo membrane damage induced by live *Staphylococci*. Therefore, injection with heat-killed bacteria also led to diminished Sqstm1 recruitment and possible xenophagy. Observations by Mitchel *et al*. reveal a similar phenomenon in macrophages infected by *Listeria monocytogenes*, where bacteria were initially encapsulated within LC3-positive phagosomes, and subsequent damage of the vacuoles triggers xenophagy [51]. In conclusion, our results suggest a dual role for the autophagy machinery in neutrophils, with the formation of LAPosomes facilitating the intracellular life stage of *S. aureus* and the Sqstm1-dependent mechanism providing partial protection. This mechanism could be inducing xenophagy, but other roles of Sqstm1 have been previously observed, such as a signaling function in inflammasome formation [52] or controlling the mechanism of programmed cell death [53].

**Figure 10.**
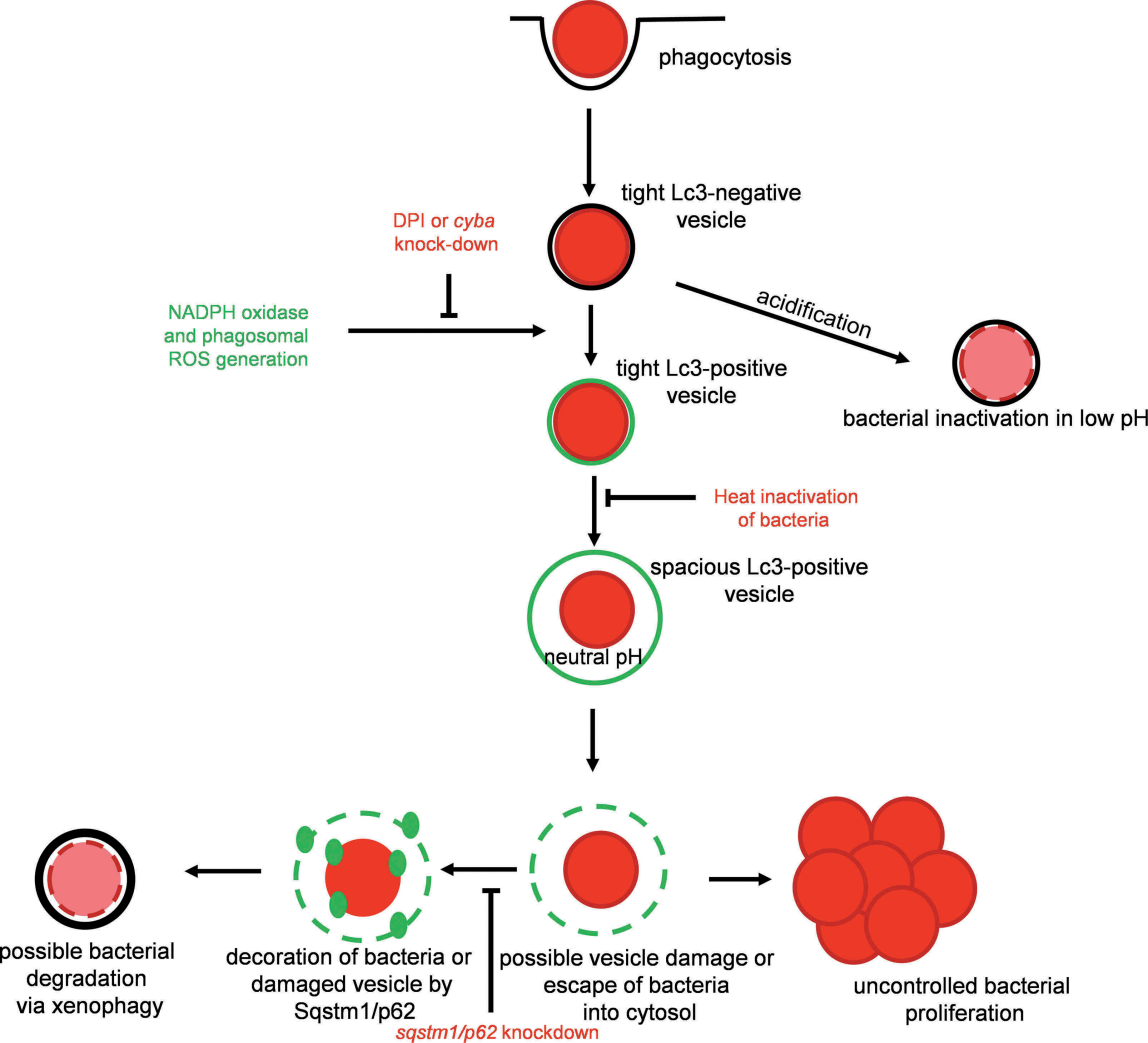
Proposed model of fate of *S. aureus* within neutrophils. Staphylococci are internalized by neutrophils and trapped within LC3-associated phagosomes (LAPosomes) triggered by NADPH oxidase. LAPosomes do not get acidified and provide a replication niche for internalized *S. aureus*, which eventually will damage the phagosomal membrane and escape into the cytoplasm. This is sensed by Sqstm1/p62 – a member of the selective autophagy machinery, which could lead to the formation of autophagosomes containing staphylococci and leading to pathogen inactivation

Therefore, we propose the following model of the fate of staphylococci within neutrophils (Figure 10). Bacteria are internalized by neutrophils and trapped within LC3-associated phagosomes which are triggered by phagosomal NADPH oxidase. These LAPosomes do not get acidified, allowing internalized *S. aureus* to damage the phagosomal membrane. Bacteria or damaged phagosomal membrane may then be detected by the selective autophagy receptor protein Sqstm1, which might lead to the formation of autophagosomes containing staphylococci and pathogen inactivation. The inability to sequester all bacteria from damaged LAPosomes or inhibition of autophagy flux could lead to subsequent bacterial dissemination. Together, the observed antagonistic role of the autophagic machinery (LAP vs. Sqstm1-mediated response) within *S. aureus*-infected neutrophils may explain the conflicting reports on anti-staphylococcal autophagy. Clarification of the molecular mechanisms of how *S. aureus* is engaged in LAP awaits future study, which may provide new insights for therapeutic strategies to fight this intracellular pathogen.

## Materials and methods

### Zebrafish lines and maintenance

Zebrafish adults and embryos were handled in compliance with local animal welfare regulations and maintained according to standard protocols (zfin.org) in compliance with international guidelines specified by the EU Animal Protective Directive 2010/63/EU. Existing lines were London wild-type (LWT), *Tg(CMV:EGFP-map1lc3b)zf155* [27] and *Tg(lyz:GFP-sqstm1/p62)i330* [40]. For the generation of the *Tg(lyz:RFP-GFP-map1lc3b)sh383* line, zebrafish RFP-GFP-Lc3 middle entry clone [34] and pDestTol2CG [54] was used to generate pDEST(*lyz*:RFP-GFP-Lc3). Embryos of wild type zebrafish lines (LWT) were injected with a transgenic expression construct together with the *tol2* transposase RNA generated as previously described [54]. Positive embryos were selected for mosaic expression under a Leica MZ10 F fluorescence dissecting microscope (Leica), by GFP heart marker. Selected embryos were raised to adulthood, and after 3 months, sexually mature fish were screened by outcrossing to LWT fish. The offspring of potential founders were screened for transgene expression. Embryos were incubated in E3 medium (5 mM NaCl, 0.17 mM KCl, 0.33 mM CaCl_2_, 0.33 mM MgSO_4_) at 28.5°C according to standard protocols [55].

### Bacterial cultures and infection experiments

*Staphylococcus aureus* SH1000 expressing mCherry (SH1000 pMV158-mCherry) [28] was cultured in brain heart infusion (BHI) broth medium (Sigma, 53286) at 37°C supplemented with tetracycline (Sigma, 87128) at 5 µg/ml. Zebrafish larvae at 30 hpf (hours post-fertilization) were microinjected into the circulation with bacteria as previously described [25]. Briefly, anesthetized larvae were embedded in 3% w/v methylcellulose (Sigma, M7027) and injected individually using microcapillary pipettes (WPI, TW100-4) filled with the bacterial suspension of known concentration. For macrophage depletion, clodronate liposomes (Liposoma BV) were injected at 26 hpf, as previously described [38]. Following infection, larvae were observed frequently up to 120 hpf, and numbers of dead embryos recorded at each time point.

### Determination of in vivo bacterial loads

At various times post-infection, living zebrafish larvae were anesthetized and individually transferred with 100 µl of E3 medium into 0.5 ml tubes containing 1.4 mm ceramic beads (Qiagen, 13113) and homogenized using a Precellys 24-Dual homogenizer (Peqlab). The homogenates were serially diluted and plated on BHI agar to determine *S. aureus* CFU numbers. Bacterial load was also determined for dead larvae at each time point.

### Morpholino knockdown and RT-PCR for morpholino efficacy verification

Morpholino oligonucleotides (Gene Tools) were dissolved in MilliQ water to obtain the required concentrations. 1 nl volume of morpholino was injected into the yolk of 1–4 cell stage zebrafish embryos using a microinjector. Standard control morpholino (Gene Tools) was used as a negative control. The *irf8* [30], *cyba* [37], and *sqstm1*/*p62* [41] morpholinos were used at the previously published concentrations. The *sqstm1* knockdown was verified by RT-PCR with a pair of primers flanking the splicing event between the first intron and the second exon (i1e2) as described previously [41].

### CRISPR-mediated knockdown of atg5 and atg16l1

The online web tool CHOPCHOP was used to design a specific guide RNA in exon 3 of *atg5* and exon 2 *atg16l1*. Purified crRNA, Cas9 protein, and tracrRNA were purchased from Sigma-Aldrich. We used the following crRNA sequences, where the PAM site is indicated in brackets: *atg5*: TCAGGTAACTGACCCGTGGG(AGG) *atg16l1*: TTTGTGGAAGCGTCACGTTG(TGG). Each embryo was injected with 1 nl of 16.6 µM crRNA, tracrRNA, and Cas9 protein mixture at the one-cell stage. As controls, only Cas9 + tracrRNA were without crRNA.

### Treatment with diphenyleneiodonium (DPI)

At 1 h before infection, embryos were treated with 100 µM DPI (Sigma, D2926) in E3 medium. Embryos were infected and kept in DPI for imaging experiments, or in the case of survival experiments, until 28 hpi.

### TSA staining

Infected embryos were fixed in ice-cold 4% (w:v) paraformaldehyde (PFA, Thermo Scientific, AAJ19943K2) in PBS-TX (PBS [Sigma, 18912014] supplemented with 0.5% Triton X-100 [Sigma, X-100]) overnight at 4°C. Fixed embryos were washed in PBS-TX twice. Peroxidase activity was detected by incubation in 1:50 Cy5-TSA:amplification reagent (PerkinElmer, NEL745E001KT) in the dark for 10 min at 28°C followed by extensive washing in PBS-TX.

### Staining of S. aureus with pH-sensitive dyes

The pHrodo Red (Life Technologies, P36600) and Fluorescein-5-EX (Life Technologies, F6130) S-ester dyes were dissolved in DMSO (Sigma, D8418) to the final concentrations of 2.5 mM and 16.95 mM, respectively. 0.5 μl of pHrodo Red and 1.5 μl Fluorescein was added to 200 μl of bacterial suspension in PBS pH 9 and then mixed thoroughly. The mixture was incubated 30 min at 37°C with gentle rotating. To remove the excess of the dyes, bacteria were washed during 3 step procedure: addition of 1 ml of PBS pH 8, 1 ml of Tris pH 8.5, again 1 ml of PBS pH 8, followed by 2 min of centrifugation in 12000 g and gentle removal of the supernatant. After washing, bacterial pellet was resuspended in 200 μl of PBS pH 7.4 and proceeded to microinjections of zebrafish embryos.

### Imaging and Image analysis

Live anesthetized or PFA-fixed larvae were mounted in 1% (w:v) low-melting-point agarose (Sigma, A4018) solution in the E3 medium. For live larvae, images were acquired using the UltraVIEW VoX spinning disk confocal microscope (Perkin Elmer) with Olympus 40x UPLFLN oil immersion objective (NA 1.3). For fixed samples, images were acquired using Leica TCS SPE laser scanning confocal microscope with a 63x HC PL APO water immersion objective (NA 1.2).

For quantification of the autophagic response within infected phagocytes, for each embryo, a total number of observable infected phagocytes were manually determined through the z-stacks of acquired images. Among these total observable infected phagocytes number of infected phagocytes with GFP-Lc3 signals were enumerated, and the percentage of Lc3-positive phagocytes over total observable phagocytes was determined for each embryo. Maximum projections were used for representative images. No non-linear normalizations were performed.

### Statistical analysis

Survival experiments were evaluated using the Kaplan-Meier method. Comparisons between curves were made using the Log Rank (Mantel-Cox) test. Quantifications of percent Lc3-positive phagocytes or CFU counts were determined for significance with unpaired parametric t-test for 2 groups and with ANOVA for multiple groups, corrected for multiple comparisons. Analysis was performed using Prism version 7.0 (GraphPad). Statistical significance was assumed at P values below 0.05.

## Supplementary Material

Supplemental Material
