# Supplementary material for: The autophagic response to Staphylococcus aureus provides an intracellular niche in neutrophils
Source: Autophagy. 2020 Mar 15;17(4):888–902. doi: 10.1080/15548627.2020.1739443 (PMC8078660; doi:10.1080/15548627.2020.1739443)
Supplement: Supplemental Material [file KAUP_A_1739443_SM7150.zip › Supplementary Figure Legends R3.docx]

**Figure S1.** Different kinetics of the Lc3-mediated response in macrophages and neutrophils infected by *S. aureus*. (**A** **and** **B**) Quantification of the percentage of infected neutrophils out of total neutrophils observed (**A**) and the average number of bacteria per infected neutrophil (**B**) within control and *irf8* knockdown embryos at 6 hpi with approximately 1500 CFU mCherry*-*labeled *S. aureus*. Data are shown as mean +/- standard deviation (SD) obtained from two independent experiments (7 larvae per experiment per group). We analyzed 153 neutrophils in 14 control larvae. We analyzed 508 neutrophils in 14 *irf8* knockdown larvae. Unpaired two-tailed t-test was used. *** *P*<0.001, * *P*<0.05. (**C**) Confocal photomicrographs are shown as maximum intensity projections of control (top panel) and *irf8* knockdown (bottom panel) fixed CMV:GFP-Lc3 embryos infected with approximately 1500 CFU of mCherry-labeled *S. aureus*. Embryos were fixed at 6 hpi and chemically stained for Mpx activity (TSA, magenta). The images shown are representative of three independent experiments. Scale bars:10 µm. (**D**) Quantification of Lc3 associations with intracellular *S. aureus* within neutrophils at 6 hpi of control and *irf8* knockdown larvae infected with approximately 1500 CFU. Data are shown as mean +/- standard deviation (SD) obtained from three independent experiments (6 larvae per experiment per group). We analyzed 65 infected neutrophils in 18 control larvae. We analyzed 197 infected neutrophils in 18 *irf8* knockdown larvae. Unpaired two-tailed t-test was used. ns – not significant. (**E**). A representative confocal photomicrograph of the Lc3-mediated response within a neutrophil of a live CMV:GFP-Lc3 embryo infected with approximately 1500 CFU of mCherry-labeled *S. aureus*. Neutrophils were selected for imaging based on cell morphology (smaller size than macrophages) and relatively low number of phagocytosed bacteria compared to macrophages.

**Figure S2.** Formation of NADPH oxidase-dependent Lc3-positive vesicles containing *S. aureus* in neutrophils is detrimental for the infected host. (**A**) Survival of control (DMSO) and DPI-treated *irf8* knockdown zebrafish larvae following intravenous injection with approximately 1500 CFU of *S. aureus* at 30 hpf (25 larvae per group). This result is representative of three independent experiments. Survival curves were compared using a log-rank (Mantel-Cox) statistical test. **P*<0.05. (**B** **and** **C**). Quantification of the percentage of infected neutrophils out of total neutrophils observed (**B**) and the average number of bacteria per infected neutrophil (**C**) within *irf8*-only or *irf8* + *cyba* knockdown embryos at 1 hpi with approximately 1500 CFU mCherry*-*labeled *S. aureus*. Data are shown as mean +/- standard deviation (SD). We analyzed 115 neutrophils in 7 *irf8* only knockdown embryos. We analyzed 118 neutrophils in 7 *irf8* + *cyba* knockdown embryos. Unpaired two-tailed t-test was used. ns – not significant. (**D** **and E**). Quantification of the percentage of infected neutrophils out of total neutrophils observed (**D**) and the average number of bacteria per infected neutrophil (**E**) within *irf8* knockdown embryos treated with DMSO or DPI at 1 hpi with approximately 1500 CFU mCherry*-*labeled *S. aureus*. Data are shown as mean +/- standard deviation (SD). 183 neutrophils in 7 DMSO-treated larvae were analyzed. We analyzed 145 neutrophils in 7 DPI-treated larvae. Unpaired two-tailed t-test was used. ns – not significant. (**F**) Survival of macrophage-depleted (clodronate-treated) control and *cyba* knockdown zebrafish larvae following intravenous injection with approximately 1500 CFU of *S. aureus* at 30 hpf (≥25 larvae per group). This result is representative of three independent experiments. Survival curves were compared using a log-rank (Mantel-Cox) statistical test. *** *P*<0.001. (**G**) Survival of control and *cyba* knockdown zebrafish larvae following intravenous injection with approximately 1500 CFU of *S. aureus* at 30 hpi (≥25 larvae per group). This result is representative of three independent experiments. Survival curves were compared using a log-rank (Mantel-Cox) statistical test. ns – not significant.

**Figure S3.** Depletion of LAP by targeting Atg proteins is beneficial to the *S. aureus*-infected host. (**A** **and** **B**) Confocal photomicrographs shown as maximum intensity projections of the Lc3-mediated response at 1 hpi in *irf8*-only (**A**) and *irf8* + *atg5 + atg16l1* knockdown (**B**) live *lyz*:RFP-GFP-Lc3 embryos infected with approximately 1500 CFU of mCherry-labeled *S. aureus*. Scale bars: 10 µm. (**A’** **and** **B’**). Zoomed-in fragments of photomicrographs. (**C**) Quantification of Lc3 associations with intracellular *S. aureus* within infected neutrophils of *irf8*-only and *irf8* + *atg5 + atg16l1* knockdown *lyz*:RFP-GFP-Lc3 embryos at 1 hpi with approximately 1500 CFU. Data are shown as mean +/- standard deviation (SD) obtained from three independent experiments (6-8 larvae per experiment per group). We analyzed 235 infected neutrophils in 24 *irf8*-only knockdown larvae. We analyzed 197 infected neutrophils in 25 *irf8* + *cyba* knockdown larvae. Unpaired two-tailed t-test was used. **** *P*<0.0001. (**D**) Survival of *irf8*-only or *irf8* + *atg5* + *atg16l1* knockdown zebrafish larvae following intravenous injection with approximately 1500 CFU of *S. aureus* at 30 hpf (52 larvae per group). This result is representative of three independent experiments. Survival curves were compared using a log-rank (Mantel-Cox) statistical test. * *P*<0.05. (**E**) Quantification of neutrophils with spacious *S. aureus*-containing phagosomes at 1 hpi within live *lyz*:RFP-GFP-Lc3 *irf8* knockdown embryos infected with approximately 1500 CFU of live mCherry-labeled *S. aureus*. Data are shown as mean +/- standard deviation (SD) obtained from three independent experiments. We analyzed 124 infected Lc3-positive neutrophils in 18 larvae injected with live bacteria.

**Figure S4.** Loss of Sqstm1 leads to increased susceptibility to *S. aureus* infection. (**A**) Electrophoresis gel scan of reverse transcription-polymerase chain reaction (RT-PCR) products of control and *sqstm1* knockdown zebrafish at 48 hpf used to determine the efficacy of *sqstm1* splice morpholino. (**B** **and** **C**) Quantification of the percentage of infected neutrophils out of total neutrophils observed (**B**) and the average number of bacteria per infected neutrophil (**C**) within the control or *sqstm1* knockdown embryos at 1 hpi with approximately 1500 CFU mCherry*-*labeled *S. aureus*. Data are shown as mean +/- standard deviation (SD). We analyzed 46 neutrophils in 6 control embryos. We analyzed 37 neutrophils in 6 *sqstm1* knockdown embryos. Unpaired two-tailed t-test was used. ns – not significant.
