# Supplementary figures and images for: The autophagic response to Staphylococcus aureus provides an intracellular niche in neutrophils
Source: Autophagy. 2020 Mar 15;17(4):888–902. doi: 10.1080/15548627.2020.1739443 (PMC8078660; doi:10.1080/15548627.2020.1739443)

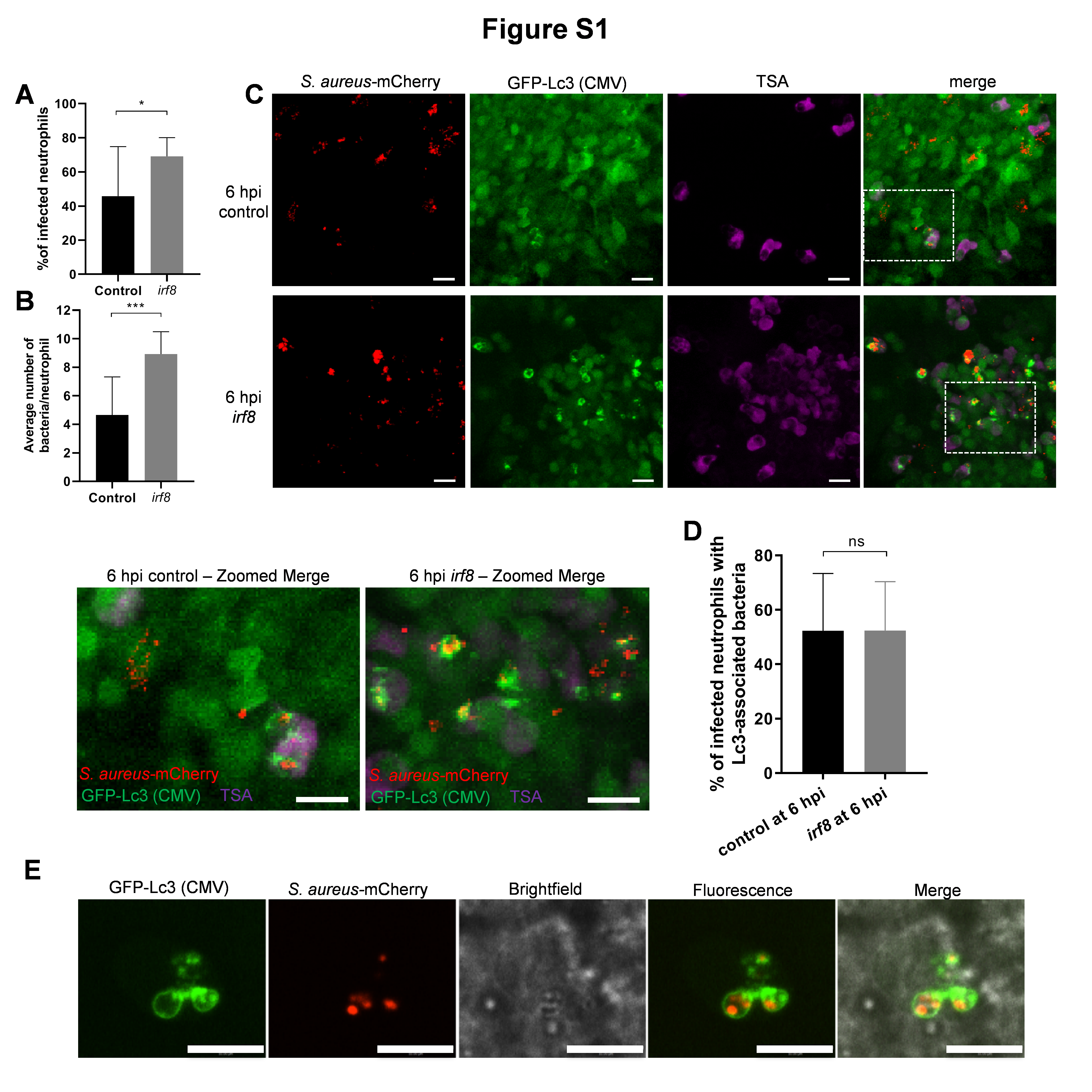

Supplement: Supplemental Material [file KAUP_A_1739443_SM7150.zip › Figure S1.tif]

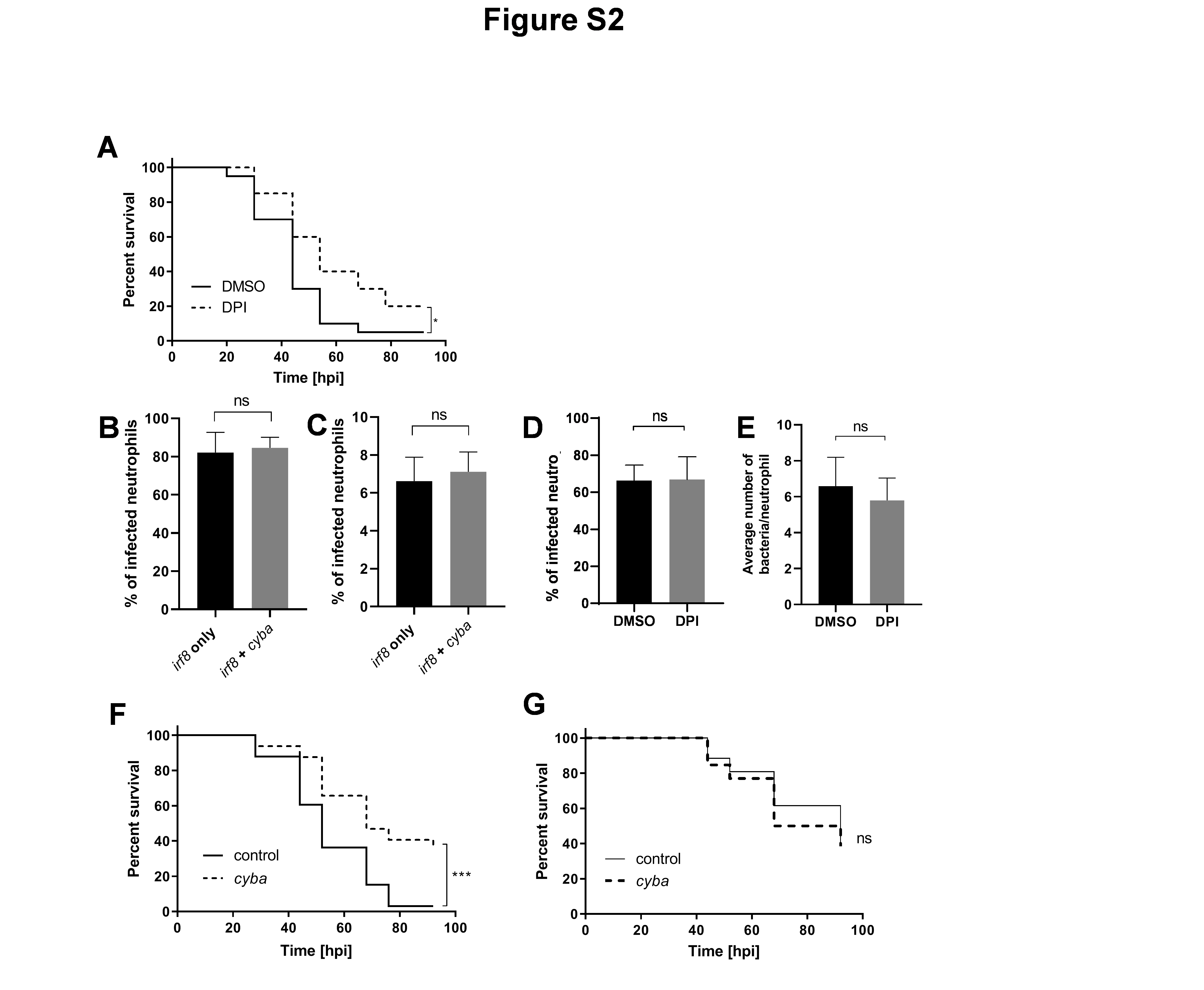

Supplement: Supplemental Material [file KAUP_A_1739443_SM7150.zip › Figure S2.tif]

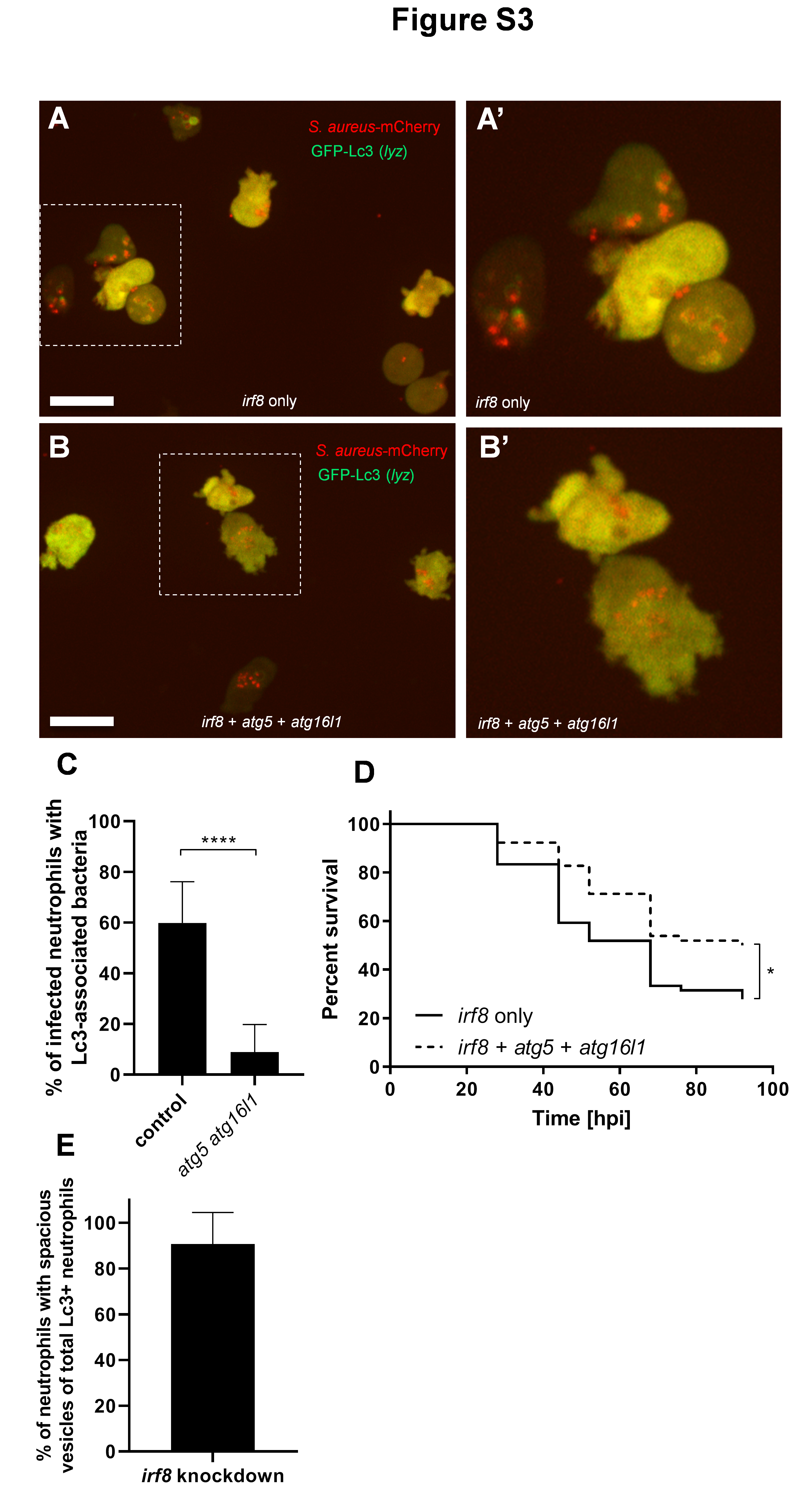

Supplement: Supplemental Material [file KAUP_A_1739443_SM7150.zip › Figure S3.tif]

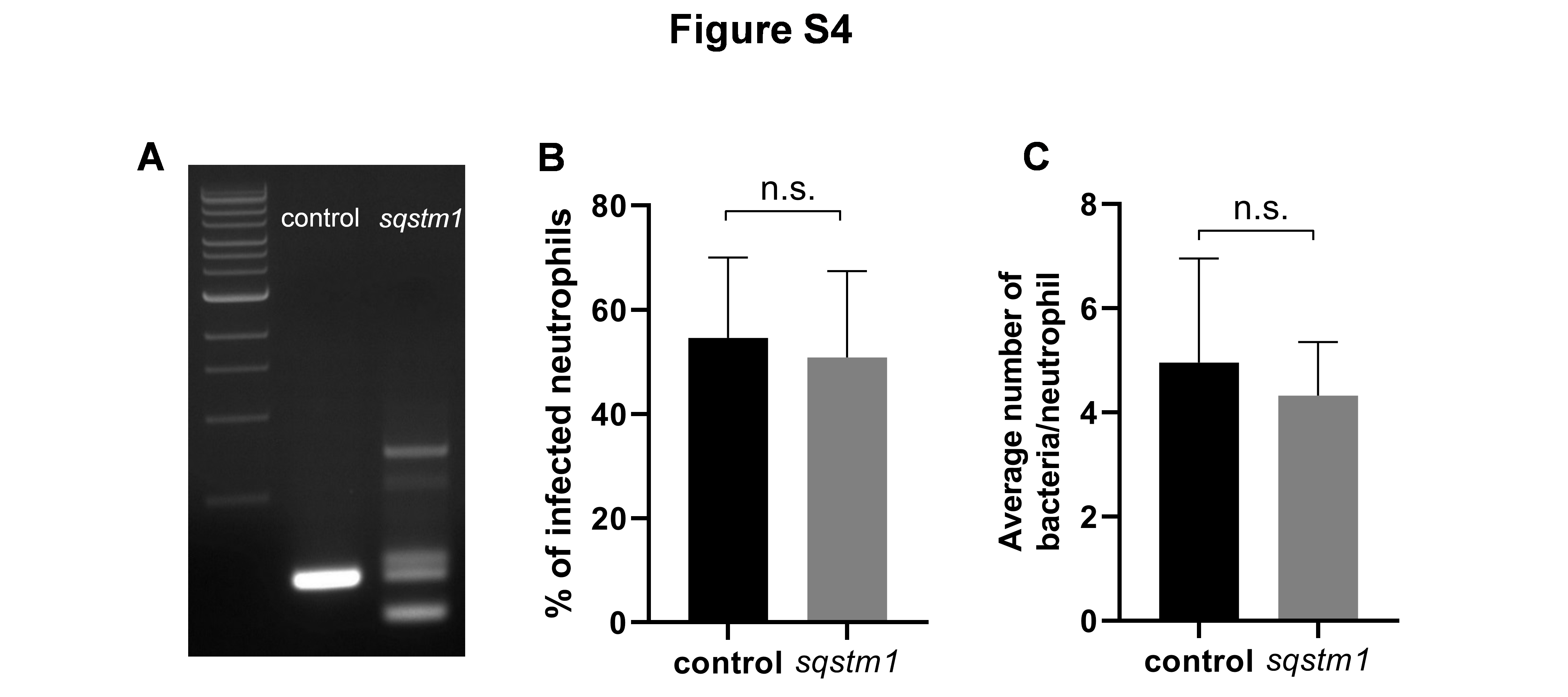

Supplement: Supplemental Material [file KAUP_A_1739443_SM7150.zip › Figure S4.tif]
